# Genomic Validation of PERV‐C‐Free Pigs to Support Xenotransplantation

**DOI:** 10.1111/xen.70109

**Published:** 2026-01-16

**Authors:** Neal R. Benjamin, Giovanni Madrigal, Yasuko Ishida, Julian Catchen, Kari L. Allen, Brent Pepin, Alfred L. Roca

**Affiliations:** ^1^ The Program in Ecology, Evolution and Conservation Biology University of Illinois Urbana‐Champaign Urbana Illinois USA; ^2^ Department of Evolution, Ecology, and Behavior University of Illinois at Urbana‐Champaign Urbana Illinois USA; ^3^ Department of Animal Sciences University of Illinois Urbana‐Champaign Urbana Illinois USA; ^4^ Cytotheryx, Inc. Rochester Minnesota USA; ^5^ National Pork Board Des Moines Iowa USA

## Abstract

Porcine endogenous retroviruses (PERVs) are present in the germ lines of domesticated pigs (*Sus scrofa*) and related suids. There are three types of PERVs, PERV‐A, ‐B, and ‐C, which differ in their host range. PERV‐A and ‐B can infect human and porcine cells, while PERV‐C only infects porcine cells. PERV‐A and ‐B are found in the genomes of all pigs, while PERV‐C is found in most but not all pigs. Although many PERV provirus insertions are defective, in vitro culture of porcine cells has produced infectious virions of all three types as well as PERV‐A/C recombinants, which show enhanced replication competence. Identifying pigs that are PERV‐C negative could help prevent such recombination events and would advance the development of porcine germplasm as a safer source of xenografts for humans. Here, we present the results of extensive screening involving 142 Landrace, Duroc, Large White, and crossbred pigs using up to nine primer pairs to identify putative PERV‐C‐negative animals. Long‐read whole genome sequencing was conducted on a subset of four pigs (one PERV‐C PCR positive and three PERV‐C PCR putative negatives), which confirmed their status as PERV‐C positive or negative, respectively. Our results confirmed that the screened pigs were truly PERV‐C negative, establishing the existence of PERV‐C‐negative germplasm within the herd. These findings support the feasibility of developing or selecting PERV‐C‐negative pigs as a source of germplasm for xenotransplantation and other biomedical applications.

## 1 Introduction

Globally, the gap between patient demand for transplantable solid organs and a limited available supply results in the deaths of thousands of people each year [1]. Xenotransplantation has the potential for providing transplantable solid organs, i.e., xenografts, without reliance on human donors. Domestic pigs (*Sus scrofa*) are suitable donor animals because of their availability, ease of care, rapid reproductive rates, large average litter sizes, and physiological similarity to humans [2]. Despite recent progress [3], several key challenges must be resolved before xenografts can be used in clinical settings. One impediment to using porcine‐derived xenografts is the presence of porcine endogenous retroviruses (PERVs) in the genomes of all domestic pigs. While most pathogens of concern can be eliminated through techniques such as aseptic removal of piglets via cesarian section, colostrum deprivation, segregated rearing, and targeted therapeutics, PERVs are permanently integrated into the genomes of their hosts and thus require more sophisticated approaches to elimination [4, 5].

Endogenous retroviruses (ERVs) are viral sequences that have integrated into the germline genome of a host species and are inherited in a Mendelian fashion. These elements are remnants of ancient retroviral infections and can make up a significant portion of the host genome. PERVs are endogenous gamma retroviruses present in the germ lines of domestic pigs and related suids. There are three types: PERV‐A, ‐B, and ‐C, which differ in their receptor‐binding domain and, thus, viral tropism [6]. While many endogenous retroviruses contain indels or other mutations that render them non‐functional, some PERVs retain the capability for active infection and reintegration. PERV‐A and PERV‐B are capable of actively infecting human cells in vitro and are found in the genomes of all pigs [7]. PERV‐C is ecotropic, i.e., only capable of infecting porcine cells, and is found in the genomes of most, but not all pigs [7].

PERV‐A/C recombinants appear to arise frequently in living swine, having been isolated as proviral integrants from somatic cells such as peripheral blood mononuclear cells, as well as multiple organs of miniature swine [2, 8, 9]. Pal et al. detected PERV‐A/C recombinant RNA in 45 of 204 pigs sampled from three commercial swine farms in the United States [10]. This suggests that such recombination may be common in individual animals with normal immune function. Nonetheless, transcriptional activation would likely be required in cases where such recombination takes place. In some cases this may happen secondarily to infection with other viruses. Such recombinants have been shown to be capable of infecting human cells and have also been detected in cultures containing both PERV‐A and PERV‐C. They are characterized by a higher replication capacity than either progenitor type, particularly after serial passage [2, 11, 12]. While PERV‐A/C recombinants have not been reported in germline cells, the increasing use of somatic cell nuclear transfer to produce pigs in high‐health barrier facilities presents a potential mechanism for their inadvertent introduction into germline cells [13]. Because cloning generates an entire organism from somatic cells, it carries the risk of unintentionally introducing PERV‐A/C recombinants into the germline of pig lineages. Therefore, more extensive screening for PERV‐A/C recombinants is warranted when screening germplasm from cloned animals or animals with clonally derived ancestors.

Considering the possible risks posed by PERV‐A/C recombinants, Bartosch et al. [12] and Denner et al. [11, 14, 15, 16] have recommended that pigs being considered for xenograft harvesting should be free of PERV‐C proviral integrants. Genome‐wide inactivation of PERVs using CRISPR‐Cas9 and related technologies is a promising alternative [17, 18]. Nonetheless, to produce xenografts for clinical use, additional modifications will be necessary (e.g., to overcome immune‐mediated incompatibilities) [3]. Additionally, the use of multiple guide RNAs is known to increase the risk of off‐target mutations regardless of the specific approach utilized [19].

While full PERV knockouts using CRISPR‐Cas9 represent a major advance for xenotransplantation, pigs that are naturally free of PERV‐C remain valuable for several reasons. Firstly, because they do not require gene editing, they avoid potential off‐target effects and may simplify regulatory approval. These animals can also serve as unmodified recipients for edited embryos or donor cells, avoiding additional editing in the host. Furthermore, when generating fully PERV‐inactivated lines, starting with PERV‐C‐free animals reduces the total number of proviral targets, making editing more efficient and manageable.

The total reported copy number of integrated PERV proviruses per cell (i.e., including all three types) has varied greatly, ranging from 3 to 117, depending on the methods used for estimation and the specific tissue being analyzed [14, 20]. There is likely variation in copy number between individuals, breeds, and even tissues within the same animal. However, some of this reported variation may be an artifact of the methods used rather than representing actual differences in copy number [14].

Overall, studies have suggested that typical germline cells may contain several dozen full‐length or near full‐length integrants across all three PERV classes. Groenen et al. reported 24 nearly complete PERV integrants in the draft whole genome reference assembly Sscrofa10.2 [21]. Chen et al. used four bioinformatics approaches to analyze whole genome sequencing data from 63 pigs, including the reference assembly Sscrofa11, an updated version of the genome examined by Warr et al. [22]. They reported a mean copy number of 32.0 ± 4.0 among Chinese breeds of pigs compared to 49.1 ± 6.5 among Western breeds [23]. Interestingly, while Chen reported 36 complete or nearly complete PERVs within Sscrofa11.1 [22], Kono et al. identified 27 full‐length PERV loci (15 PERV‐A and 12 PERV‐B) within the same swine reference genome (Sscrofa11.1) [21, 24], illustrating that genome quality and/or the specific bioinformatics approaches used may impact the ability to detect PERV copy numbers. Additionally, Kono et al. identified 27 full‐length PERV sequences, 18 PERV‐A, and 9 PERV‐B, in genome sequence data from the cell line PK15 [24]. By comparison, Luhan et al. identified 62 copies of PERVs in PK15 cells using droplet digital polymerase chain reaction (PCR), an indirect way of measuring copy number [17]. However, given that PK15 cells are immortalized kidney cells, these results may not represent the copy number found in typical somatic or germ‐line cells in animals with fully competent immune systems and typical life histories. Similarly, Niu et al. [18] detected 24 copies of PERVs (10 copies of PERV‐A, 14 copies of PERV‐B, and 0 copies of PERV‐C) using droplet digital PCR and whole genome sequencing in a primary porcine fibroblast cell line being used to generate CRISPR‐Cas9 PERV knock‐out pigs. More research is needed to confirm these findings and determine to what extent there is variation among breeds, individuals, and tissue types when comparing PERV copy counts.

The prevalence of PERV‐C across pig populations has been examined by a small number of studies, some of which may have underestimated the true prevalence of PERV‐C within the test population by limiting the number of primers used to detect PERV‐C. Wu et al. [25] found a 30.5% PERV‐C test positive prevalence (equivalent to a negative test rate of 69.5%) among 348 genomic samples from seven breeds of Chinese miniature pigs. Notably, they used the PCR1 primers originally designed by Takeuchi et al. [26] and no other primer pairs. Similarly, Pal et al. identified 48 PERV‐C‐positive animals among 204 commercial swine sampled across three herds [10]. They used a real‐time PCR assay consisting of a single pair of primers and a probe specific to PERV‐C. Using two PERV‐C‐specific primer pairs, Dieckhoff et al. [27] identified 176 PERV‐C positives among 181 samples from a mixed population of German purebred swine and transgenic animals of crossbred origin.

In this study, we sought to more comprehensively evaluate the PERV‐C status of a herd of swine by using multiple primer pairs [26, 28] to assess PERV‐C status, and to further confirming their PERV‐C negative status by genomic sequencing. Animals originated from a high‐health barrier herd, which undergoes routine pathogen monitoring as part of standard herd health management. Additional targeted viral testing (e.g., porcine cytomegalovirus/porcine roseolovirus) was not part of the present study. Animals within this herd are being developed as a porcine model system with the goal of repopulating their livers with human hepatocytes for use in preclinical drug trials or possibly for production of human hepatocytes for therapeutic uses [29]. To evaluate the presence of PERV‐C‐negative individuals within this high‐health barrier herd, we screened selected animals using multiple PCR tests [26, 28] and confirmed findings in a subset using long‐read whole genome sequencing.

## 2 Materials and Methods

### 2.1 Animal Source and Sample Collection

All swine were kept by Cytotheryx (Rochester, MN, USA), a biotechnology company focused on providing high‐quality human hepatocytes for research and cell‐based therapies. Samples were collected as part of their routine health surveillance program. All foundation germplasm procured by Cytotheryx was originally purchased from a commercial swine breeding stock company that is no longer operational. The study population comprised purebred Landrace, Duroc, and Yorkshire (Large White) pigs as well as crosses among these breeds.

As an initial filter to identify potential PERV‐C‐negative individuals, 121 animals were sent to the University of Minnesota's Veterinary Diagnostic Lab (UMN VDL), which screened them using quantitative PCR (qPCR) with for PERV‐C using primers published by Martina et al. [30] (Dr. Stephanie Rossow, personal communication, 07/19/2022). This assay was only used in this initial external qPCR screen, and these primers were not employed in our subsequent PCR assays. From these 121 individuals, 18 putatively negative animals were randomly chosen from a total of 33 putative negatives and used for further screening. A commercially available PCR screen for PERVs was subsequently offered by the Iowa State University (ISU) VDL using unpublished primers; we, therefore, note that we did not use this test to screen any of the germplasm presented here.

The study was conducted following University of Illinois Institutional Animal Care and Use Committee protocols 24120 and 21159. We extensively screened 142 porcine samples from live animals and frozen germplasm including fibroblasts (hereafter referred to as “individuals” or “animals” regardless of germplasm type unless otherwise specified). Any blood samples were collected in purple top EDTA tubes and stored at 4°C until further processing. A subset of samples was provided as previously extracted whole genomic DNA and thus eligible for further PCR testing after DNA quantification. Due to the limited availability of some samples, not all individuals were eligible for all tests.

### 2.2 DNA Extraction, Quantification, and PCR Screening

DNA extractions were performed after centrifugation with a DNeasy Blood & Tissue Kit (Qiagen, Hilden, Germany) according to the manufacturer's instructions. For the blood samples, buffy coats obtained by centrifugation were used. DNA quantification was performed using a Qubit 2.0 Fluorometer (Life Technologies Corp., CA, USA). Approximately, 50–100 ng of whole genomic DNA was used as a template for all further PCR reactions.

PCR mixes used a final concentration of 0.4 µM of each primer, 2 mM MgCl_2_, 200 µM of each dNTP (Life Technologies Corp., CA, USA), 1 µg/µL final concentration of recombinant bovine serum albumin (BSA, New England BioLabs Inc.) with 0.04 units/µL final concentration of AmpliTaq Gold DNA Polymerase (Applied Biosystems, Foster City, CA, USA). We used up to eight previously published pairs of primers to screen each sample for PERV‐C: PCR1 [26], PCR1 new variant [28], PCR2 [31], PCR3 [31], PCR4 [27], PCR5 [31], PCR6 [28], and PCR7 [28] following the nomenclature developed by Kaulitz et al. [28, 31]. For a thorough review of the development of these primers, see Jhelum et al. [32]. Further, all samples were also tested with primers designed to amplify the porcine housekeeping gene *GAPDH* [17] as a control to rule out PCR inhibition or other issues with template DNA quality. All reactions were performed using MiniAmp Plus Thermal Cyclers (Thermo Fisher Scientific, Waltham, MA, USA).

We adapted a published “step‐down” PCR protocol [33] originally designed for use with AmpliTaq Gold DNA Polymerase (Applied Biosystems, Foster City, CA, USA) to the published PERV‐C primer sets described previously. The thermal cycling conditions consisted of an initial denaturation and activation step at 95°C for 9 min and 45 s, followed by cycles of 20 s denaturation at 94°C and 30 s annealing at 60°C (first three cycles), then stepwise decreases of 2°C in four consecutive steps (58°C, 56°C, 54°C, and 52°C; five cycles each), followed by 22 cycles at 50°C, and a 1‐minute extension at 72°C, concluding with a final 7‐minute extension at 72°C. With each round of PCR, we included a no‐template control using molecular‐grade deionized water and a positive control using DNA extracted from pigs established as PERV‐C‐positive.

### 2.3 Amplicon Validation and Sanger Sequencing

By using multiple primer pairs and visualizing PCR products on agarose gels stained with ethidium bromide under UV light, we categorized animals as putatively positive or negative based on band presence and size. We used Sanger sequencing to confirm band identity in positive animals, reducing the risk of false positives from non‐specific amplification. To eliminate residual primers and unincorporated dNTPs, the amplicons were treated with Exonuclease I and shrimp alkaline phosphatase (both from USB Corporation, OH, USA) prior to the sequencing reactions [34]. Sanger sequencing was performed in both directions using the BigDye Terminator v3.1 Cycle Sequencing Kit (Applied Biosystems, Foster City, CA, USA) using 5.275 µL molecular‐grade deionized water, 1.875 µL 5× buffer, 0.25 µL Big Dye Terminator, 2 µL of purified PCR product, and 0.12 µM primer (forward or reverse) for each primer pair as previously described [35]. Products were purified and resolved on an ABI 3730XL DNA Analyzer at the DNA Services Core in the Roy J. Carver Biotechnology Center at the University of Illinois. All sequences obtained by Sanger sequencing were aligned and visualized using Sequencher v5.4.6 (Gene Codes Corporation, Ann Arbor, MI, USA) and compared to reference sequences representing the three PERV subtypes: PERV‐A (GenBank accession no. AY099323.1) [12] PERV‐B (AY099324.1) [12], and PERV‐C (AF038600) [26].

### 2.4 Whole Genome Sequencing and Bioinformatic Validation

To validate our results, four animals (#610 a PERV‐C putative negative Landrace boar, #1098 a PERV‐C putative negative crossbred sow, #710 a PERV‐C positive Landrace boar, and #892 a PERV‐C putative negative crossbred sow) were submitted for long‐read whole genome sequencing at approximately 15× coverage. High molecular weight (HMW) DNA extraction and SMRTBell library construction were completed by the Roy J. Carver Biotechnology Center at the University of Illinois. Genomic DNA was extracted using the Qiagen MagAttract HMW DNA Kit (Qiagen, Hilden, Germany). The DNA was sheared to an average fragment size of 15 kilobasepairs (Kb) using the Diagenode Megaruptor 3 (Diagenode, Liège, Belgium). Size selection was performed on the Sage BluePippin (Sage Science) using 0.75% agarose cassettes and the S1 marker (PAC‐20 kb). Libraries were prepared using the PacBio SMRTBell Prep Kit 3.0 (Pacific Biosciences of California, Inc., Menlo Park, CA, USA) and the Barcoded Overhang Adaptor Kit 8B (Pacific Biosciences of California, Inc.). Sequencing was conducted on the PacBio Revio System with 30‐h movie times, utilizing two Revio SMRT Cells (Pacific Biosciences of California, Inc.) with two barcoded libraries per SMRT Cell, the Revio Sequencing Plate (Pacific Biosciences of California, Inc.), and the Revio Polymerase Kit (Pacific Biosciences of California, Inc.).

We calculated the necessary level of genome coverage to indicate that the four pigs examined were sequenced sufficiently to reliably determine the presence or absence of PERV‐C. Briefly, assuming a binomial distribution, to achieve a 99% probability of detecting at least one read covering a heterozygous locus, assuming each read has a 50% chance of containing the locus, a minimum of seven reads is required. This is broadly in line with prior modeling which suggests that 8× coverage should be sufficient to detect a heterozygous locus approximately 90% of the time [36].

To obtain a diagnostic sequence for PERV‐C, consensus sequences were generated for all three PERV types using NCBI accessions AF435967.1, AJ133817.1, AY099323.1, HQ540591.1, HQ540592.1, Y12238.1, AY099324.1, HQ536009.1, HQ536011.1, Y12239.1, HQ536013.1, HQ536015.1, HQ536016.1, and AF038600.1, using sequences encoding the envelope protein component of the PERV sequence. We took the set of PERV‐A sequences and created an alignment using MAFFT v7.310 [37]. The alignment was then used to generate a consensus sequence using EMBOSS cons [38]. This step was then repeated separately for both sets of PERV‐B and ‐C sequences. The three consensus sequences were then aligned to one another using MAFFT v7.310 [37] and the resulting alignment was visually inspected to identify a region unique to the PERV‐C consensus sequence and conserved among the different PERV‐C accessions. A 50 bp region uniquely conserved in PERV‐C was identified in this alignment and used to screen both genome assemblies and raw, PacBio long reads for presence or absence of PERV‐C (Figures 3A, S2, and S3). To confirm its specificity for PERV‐C, we used the 50 bp region as a query to BLASTN [39] to search the NCBI nucleotide database for matches not pertaining to PERV‐C.

For each of the four sequenced samples, the raw reads were assembled using Hifiasm v0.19.8 [40]. Klumpy v1.0.10 [41] was then employed to search for the PERV‐C diagnostic sequence across the primary and haplotype assemblies, along with the initial raw reads, setting the k‐mer size to 11 and the minimum number of k‐mers per “klump” (i.e., clusters of shared k‐mers) to 3. To discern the presence or absence of a PERV‐C across the predicted negative individuals, a PERV‐C klump in the #710 (putative PERV‐C positive) genome assembly was used to pinpoint the location of a putative PERV‐C locus. The boundaries of the locus were determined by comparing the de novo assembled sequence with PERV‐C loci in the NCBI NR database using BLASTN [39]. After establishing the locus of the putative PERV‐C sequence, the locus and the flanking 1 kilobase pairs (Kbp) regions were aligned to the negative primary Hifiasm assemblies using minimap2, version 2.24 [42]. The alignments were then examined using the Klumpy subprogram “alignment plot” to determine the presence/absence of the PERV‐C locus for each individual. Finally, the de novo assembled chromosome containing the PERV‐C locus was aligned to Sscrofa11.1 [22] to identify the corresponding reference genome chromosome.

## 3 Results

Given the potential for establishing a PERV‐C‐negative pig line through selective breeding, we investigated whether such individuals could be reliably identified using existing diagnostic methods. Our study aimed to evaluate the sensitivity of common PERV‐C diagnostic screening methods and explore whether some pigs classified as negative by PCR might in fact carry undetected proviral elements. To address this, we performed three rounds of progressively more intensive screening using a combination of PCR assays and genome sequencing.

One hundred twenty‐one animals were previously screened using qPCR at the UMN VDL using the primers described by Martina et al. [30] (Table S1). Of these, 62 (51.2%) tested positive for PERV‐C, 26 (21.5%) were classified as suspect, and 33 (27.3%) were negative. Here “suspect” corresponds to samples that resulted in cycle threshold (Ct) values ≥  36.0 (Dr. Stephanie Rossow, personal communication, July 19, 2022). Several suspect animals were later reclassified as positive after retesting. All samples were positive for PERV‐A and PERV‐B, consistent with prior literature. These results provided a baseline for our study and raised the question of whether some true PERV‐C positives might be missed by this single‐primer approach.

To evaluate the sensitivity of our internal PCR protocol and to estimate an initial prevalence of PERV‐C, we screened 28 animals using a single high‐sensitivity primer pair (“PCR1”) [26] targeting a different region of the provirus from the primer pair of Martina et al. [30]. All animals tested positive by gel visualization and/or Sanger sequencing. Among the 17 of 28 animals with prior results based on the primers of Martina et al. [30], 2 had previously been classified by the UMN VDL as negative, 4 as suspect, and 11 as positive, illustrating a clear mismatch between assays (Figure S1). These results provided a baseline for our study and raised the question of whether some true PERV‐C positives might be missed by this single‐primer approach.

**FIGURE 1:**
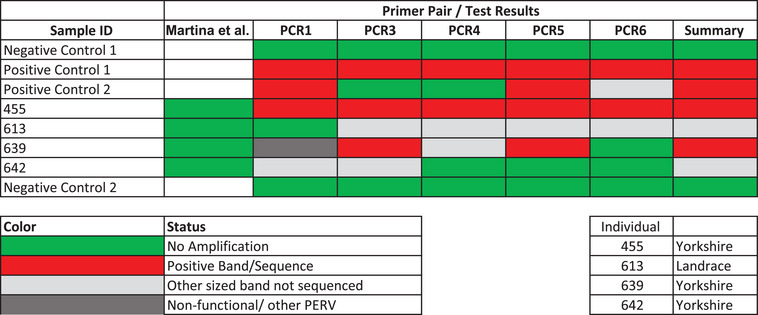
Screening for PERV‐C using five primer pairs [26, 27, 28, 31] (see Section 2) for four PERV‐C putative negative animals (numbered) along with two positive controls (established PERV‐C positive pigs) and two negative controls (no DNA). Initial putative negative status was determined by using an assay using primers developed by Martina et al. [30] (column Martina et al.), run at the University of Minnesota's Veterinary Diagnostic Lab. Red cells represent positive bands of the correct size, which were also confirmed to have amplified PERV‐C by Sanger sequencing of the resulting amplicons. Light gray cells indicate bands of incorrect size. Sanger sequencing on subset of these bands of incorrect size did not produce PERV‐C sequences. Dark gray indicates amplification of a non‐functional PERV‐C‐like integrant. White cells indicate that a sample was not tested using a specific primer pair/modality. The summary column summarizes all positive tests or visible DNA amplification of incorrect size in the case of animals that were still classified as putative negative after additional screening.

To further probe this discrepancy, we selected four animals previously classified as negative using the Martina et al. [30] primers and subjected them to a broader panel of five distinct PCR assays. These primer sets [26, 27, 28, 31] were chosen to avoid overlapping binding regions and maximize coverage of the proviral genome. Two of the four animals tested positive across multiple assays with Sanger confirmation, suggesting that prior screening had failed to detect at least some proviral insertions of PERV‐C. The other two remained PCR‐negative across all assays and were classified as putative PERV‐C negatives (Figure 1). This round demonstrated that even among pigs labeled as negative, a subset may harbor additional PERV‐C integrations detectable only by more diverse primer sets.

**FIGURE 2:**
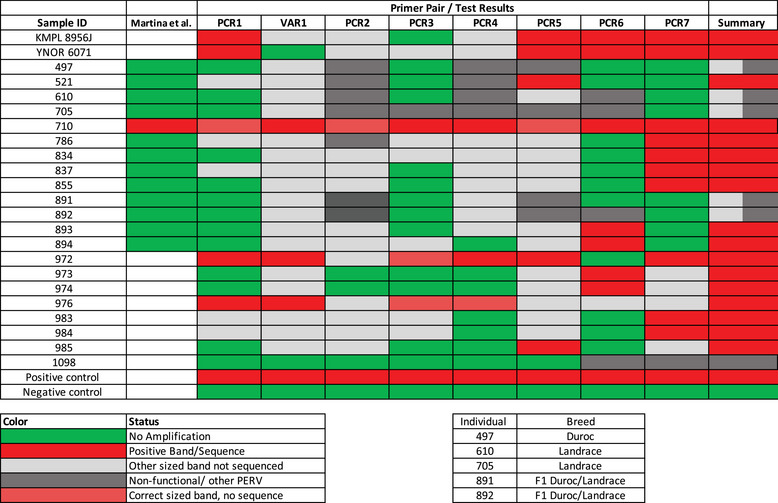
Extensive screening of 23 pigs with all 8 pairs of previously published primers for PERV‐C (and for a subset, additionally the primers developed by Martina et al. [30]). Green indicates no visible amplification, and red indicates amplification with a correct‐sized band that was also confirmed via Sanger sequencing of the amplicons. Light gray indicates amplification of incorrect size, and dark gray indicates amplification of a non‐functional PERV‐C‐like integrant. Light red bands indicate bands of the correct size that were not sequenced because the status of that individual had already been confirmed via Sanger sequencing. White bars indicate that a sample was not tested using a specific primer pair/modality. The summary column summarizes all positive tests or visible DNA amplification of incorrect size in the case of animals that were still classified as putative negative after additional screening (light and/or dark gray in the summary column). Note that pigs #497, #610, #705, #891, #892, were consistently negative for PERV‐C using all primer pairs.

To assess whether these findings were isolated or more generalizable, we screened 23 additional animals using an expanded panel of 8 primer sets. Thirteen of these animals had previously been classified as negative and one as positive using the primers of Martina et al. [30] (column Martina et al. in Figure 2). Among the 23, 6 were classified as putative PERV‐C negatives based on the absence of amplification of appropriate sized bands across all assays and/or Sanger sequencing revealing amplification of non‐PERV‐C elements (Figure 2). Five of these six putative PERV‐C negatives had previously tested negative by an assay using the primers of Martina et al. [30]. The remaining animals showed strong evidence of PERV‐C presence, often confirmed by multiple primer pairs and Sanger sequencing. These results confirmed that more comprehensive screening substantially increases detection.

Demonstrating the absence of a provirus is inherently more challenging than confirming its presence, as absence requires ruling out all potential loci rather than detecting a single positive signal. To independently validate the identification of pigs as PERV‐C negative, we sequenced the genomes of four pigs: one putative PERV‐C positive and three putative PERV‐C negatives. Our goal was to test whether the presumed absence of PERV‐C would be corroborated by analysis of genomic sequences, thereby strengthening our confidence in negative classifications. Sequencing coverage ranged from 12 to 18x. Genome assemblies for male pigs were 2.6–2.7 Gb in length, while female assemblies were 2.8–2.9 Gb. Contig N50 values ranged from 42 to 287 megabasepairs (Mb) (Supplementary Table S2).

**FIGURE 3:**
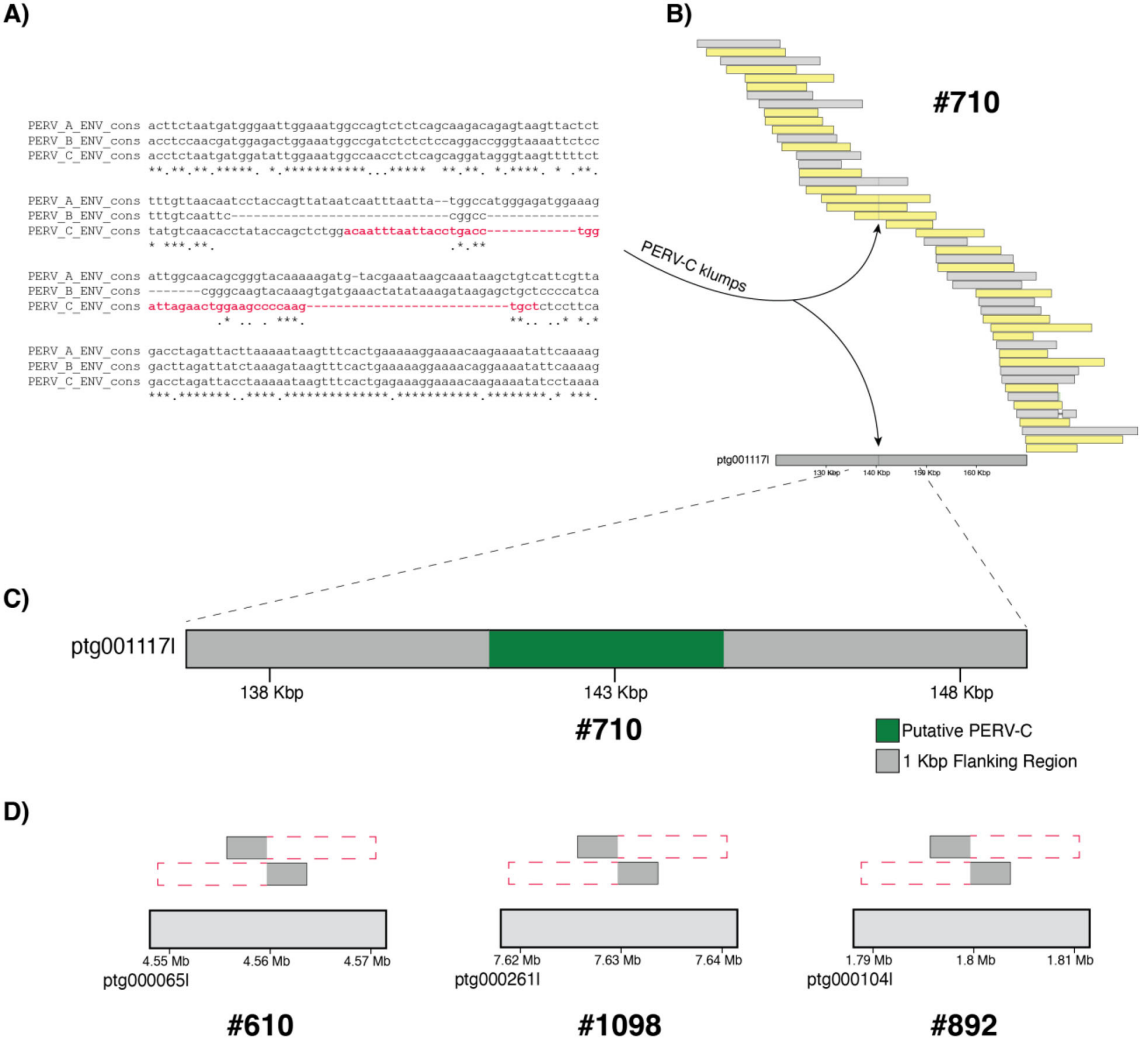
PERV‐C is absent in selected pigs: genomic evidence for a pre‐integration locus state. (A) A 50 bp region in the consensus sequence unique to PERV‐C (red colored nucleotides not present in PERV‐A nor PERV‐B) was identified and used to search for PERV‐C integrants across the four sequenced individual pigs. This sequence was chosen because it was diagnostic for PERV‐C identity. (B) An alignment plot of PERV‐C klumps in both the genome assembly and raw reads is illustrated for the PERV‐C positive pig #710. The presence of the consensus sequence in the genome verified that #710 was positive for PERV‐C. Gray bars represent alignments on the forward strand and yellow bars represent alignments on the reverse strand. (C) The PERV‐C haplotype in pig #710 and its corresponding 1 kilobase pair (Kbp) regions were identified and used to query the genome assemblies of putatively PERV‐C‐negative pigs. (D) The flanking regions around the PERV‐C integrant in pig #710 aligned to the genomes of the other pigs that were putatively PERV‐C negative, but the PERV‐C region did not align in these individuals, indicating that this locus in their genomes remained in the pre‐viral state. No other matches to the PERV‐C consensus were detected, indicating that pigs #610, #1098, and #892 did not carry copies of PERV‐C.

We identified PERV‐C in the genome of pig #710, which had been identified as PERV‐C‐positive by PCR. A cluster of 21 k‐mers matching the unique PERV‐C motif was identified on a 2.5 Mb contig in the genome of pig #710, supported by four raw reads (Figure 3B). In contrast, no PERV‐C diagnostic sequence matching clusters were found in any of the three putatively PERV‐C negative pigs.

The region flanking the PERV‐C locus in pig #710 was further identified in the three PERV‐C‐negative pigs as present but without viral sequence. To show this, we aligned the PERV‐C locus and its flanking 1 Kb regions to the genome assemblies of the three negative animals. In each case, the primary alignments were soft‐clipped (i.e., portions of the query sequence were present but did not align to the reference, which indicated structural differences at the expected integration site) by ∼1.7–3.5 Kb and contained multiple indels (Supplementary Figure S4). Secondary alignments (i.e., additional, lower‐scoring alignments of the same sequence to other genomic regions) revealed a consistent pattern of adjacent fragments flanking the expected insertion site but no continuous mapping across the full locus—strongly supporting the absence of this PERV‐C integrant in these individuals (Figure 3C,D).

In the positive pig #710 (a putative PERV‐C positive boar), the PERV‐C integrant was insertionally polymorphic, i.e., present in one but not both copies of the locus, with the other copy present not showing viral sequence. When searching for signals of PERV‐C in #710 outside the diploid, primary assembly, a 21‐mer fragment was found in one of the haplotype‐specific assemblies and in four raw reads, suggesting that at this locus this individual may be insertionally heterozygous for PERV‐C. Upon aligning the locus containing the putative PERV‐C locus in #710 to the pig reference genome Sscrofa11.1 [22], we found the locus to match a segment in chromosome 13; a chromosome that was reported to contain PERVs in other breeds [43].

After demonstrating that several of the pigs were true negatives using genomic sequencing, we considered this result in the context of PCR screening to determine the prevalence of PERV‐C‐positive and ‐negative pigs in the herd. Across three rounds of PCR testing using up to eight previously published primer pairs, we reevaluated (1) 18 animals that had previously tested negative, (2) 4 animals previously classified as suspect, and (3) 11 animals previously classified as positive using the primers of Martina et al. [30] by UMN VDL. Of 18 animals previously tested negative, 7 (38.9%, 95% CI: 17.3%–64.3%) remained classified as putative negatives, while 11 (61.1%, 95% CI: 35.8%–82.7%) were reclassified as true positives. All four animals previously reported as “suspect” were confirmed as true positives. All 11 animals previously classified as positive using the primers of Martina et al. [30] were confirmed as true positives (100%, 95% CI: 71.5%–100%). Additionally, we screened 21 animals not included in the original screening cohort and identified 20 as PERV‐C positive and 1 as a true negative confirmed via long‐read sequencing (positive rate: 95.2%, 95% CI: 76.2%–99.9%).

Assuming that suspect and positive results from initial screening reflect true positives, we estimate the true positive prevalence of PERV‐C in this herd to be approximately 89.4% (95% CI: 82.7%–93.7%, total sample size = 142). This estimate is based on the observed true negative rate of 7/18 in retested negative animals, an apparent negative rate of 33/121 in the initial screen, and the detection of 1 negative among 21 newly screened individuals. This prevalence is lower than the 97.2% reported by Dieckhoff et al., which falls outside our estimated confidence interval; however, it remains broadly consistent with their findings, given potential differences in population structure and detection methods [27].

## 4 Discussion

Our results reveal both the limitations of single‐primer PERV‐C screening and the feasibility of identifying truly PERV‐C‐negative individuals within a high‐health barrier herd using a multi‐primer PCR approach. Initial screening with the primers of Martina et al. [30] resulted in 27.3% of animals classified as PERV‐C negative. However, across subsequent rounds of testing with up to eight additional previously published primer pairs, we found that many of these negative results were likely false negatives. Specifically, 11 of 18 animals previously classified as negative were reclassified as positive using more comprehensive screening, and several suspect classifications were similarly confirmed as true positives.

This discrepancy underscores the need for multiplex or multi‐primer‐pair strategies for accurately detecting PERV‐C proviral integrations. While Jhelum et al. [32] recommend PCR1 and PCR4 as sufficient for identifying PERV‐C‐negative germplasm, our findings suggest that, at least under some conditions these alone may miss some true positives. In our dataset, PCR5 and PCR6 revealed additional PERV‐C‐positive animals that would have otherwise been misclassified, possibly due to differences in PCR conditions and polymerase choice or to primer site sequence variation. Jhelum et al. [32] noted that PCR5 and PCR6 were unreliable because, in some cases, they failed to amplify PERV‐C integrants that were amplified by PCR1 and PCR4. In several cases, we observed non‐specific bands on agarose gels but confirmed through Sanger sequencing that these bands in some cases represented a PERV‐C‐like, nonfunctional element (i.e., containing multiple missense mutations). However, in at least four cases (e.g., pigs #521, #894, #893, and #494), these primers correctly identified true positives that PCR1 and PCR4 missed.

During screening of true negative animals, DNA amplification often produced bands of unexpected sizes rather than yielding no amplification. In some cases, PCR1 and PCR2 amplified PERV‐A instead of PERV‐C. This cross‐amplification is likely due to the use of a “step‐down” PCR protocol combined with 2 mM MgCl_2_ and 1 µg/µL recombinant BSA, which enhance overall sensitivity. While these conditions maximize PERV‐C detection and help avoid false negatives, they also increase the likelihood of non‐specific amplification. To mitigate this, we recommend using PCR1 and PCR4 for initial screening, as previously suggested, followed by confirmation with additional primers to verify negative results [32]. Sequencing of unexpected bands confirmed amplification of other PERV types, unrelated genomic DNA, or non‐functional PERV‐C‐like elements.

We note that the PCR conditions used in this study were optimized to maximize sensitivity across diverse primer sets rather than to establish a streamlined diagnostic protocol. As such, the conditions were intentionally permissive and are not intended as a recommendation for routine PERV‐C screening in other laboratories. Because our primary goal was to avoid false negatives, some degree of assay‐to‐assay variability, particularly in the detection of non‐specific or PERV‐C‐like elements, should not be overinterpreted as biologically meaningful. The broader conclusions of this work rely instead on Sanger sequencing and long‐read genome validation, which together provide a robust determination of PERV‐C status despite the inherent complexity of multi‐primer PCR screening.

Long‐read sequencing served as an independent validation method, confirming the absence of PERV‐C in three PCR putative‐negative animals and identifying a PERV‐C locus in a fourth individual that was known to be positive, where one integrant was detected that was likely insertionally heterozygous. The genome assemblies for all four samples fell within the same size range as previous pig genome assemblies [22, 44, 45], and the minimum coverage for each assembly fell above the 7‐fold minimum coverage that we calculated would sequence a heterozygous insertion containing PERV‐C 99% of the time. These genomic data not only validated our PCR‐based classification of pigs regarding their PERV‐C status, but also provided positional insights by locating a potential PERV‐C locus on chromosome 13 consistent with previous reports [43].

We did not have pedigree information on most of the germplasm we analyzed. However, we know that #1098 is a daughter of #497. We identified both animals as negatives, including via long‐read whole genome sequencing in the case of #1098. Given that PERV integrants present in the germline cells are expected to follow a Mendelian inheritance pattern [46], it is not surprising that a negative animal would have a negative sire. We did not have information on the PERV‐C status of the dam of #1098.

Our results indicate that a PERV‐C‐negative population of pigs can be generated using standard breeding practices. This possibility is supported primarily by the identification of negative animals [7], but also by the presence of insertional polymorphisms and low copy number in PERV‐C in others. While gene editing remains a promising long‐term strategy for eliminating PERVs from donor lines [18], strategically breeding negative individuals from naturally PERV‐C‐negative or low copy number individuals represents a practical alternative [47] especially for generating unedited recipient animals for use in embryo transfer programs.

One recent study has shown that PERV‐C‐negative embryos can remain uninfected even when gestated in presumed positive recipients [47], though the risk of in utero transmission may not be negligible. In this context, breeding PERV‐C‐negative recipients may further enhance biosecurity. Given our estimated true positive prevalence of 89.4% in this herd, sourcing PERV‐C‐negative germplasm from commercial breeding stock companies appears feasible, particularly when paired with targeted PCR screening and/or genomic validation.

Taken together, our findings reinforce the utility of multi‐primer PCR screening as a foundation for both herd management and future xenotransplantation initiatives, but also point toward a scalable, genetics‐, and genomics‐based pathway for producing PERV‐C‐free pigs without relying solely on CRISPR‐based interventions.

## 5 Conclusion

Our findings confirm that PCR screening using multiple primer pairs is an effective strategy for identifying pigs lacking PERV‐C proviral integrations. Long‐read genome sequencing served as an independent and important validation method, confirming the accuracy of our PCR screening strategy. While routine use of long‐read sequencing may not be practical for herd‐wide surveillance, it may be particularly valuable for confirming PERV‐C‐negative status in high‐value animals, such as those intended for cloning or germplasm preservation in xenograft production pipelines. PERV‐C integrants are heritable and the detection of negative pigs and of insertional polymorphisms indicate that standard breeding could be used to generate a PERV‐C‐free populations of pigs. PERV‐C‐negative lines could be established and maintained within high‐health herds, reducing the need for additional gene editing to eliminate PERV‐C, particularly in recipient animals where such editing may be impractical.

## Supporting information




**Supporting File 1:** xen70109‐sup‐0001‐SupMat.docx

**Supporting File 2:** xen70109‐sup‐0002‐FigureS1.pdf

**Supporting File 3:** xen70109‐sup‐0003‐TableS1.pdf

## References

[1] A. Lewis , A. Koukoura , G.‐I. Tsianos , et al., “Organ Donation in the US and Europe: The Supply vs Demand Imbalance,” Transplantation Reviews 35 (2021): 10058, 10.1016/j.trre.2020.100585. 33071161

[2] J. Denner , “Porcine Endogenous Retroviruses and Xenotransplantation, 2021,” Viruses 13 (2021): 2156, 10.3390/v13112156. 34834962 PMC8625113

[3] A. Ali , M. Kurome , B. Kessler , E. Kemter , and E. Wolf , “What Genetic Modifications of Source Pigs Are Essential and Sufficient for Cell, Tissue, and Organ Xenotransplantation?,” Transplant International 37 (2024): 13681, 10.3389/ti.2024.13681. 39697899 PMC11652200

[4] H.‐J. Schuurman , “The International Xenotransplantation Association Consensus Statement on Conditions for Undertaking Clinical Trials of Porcine Islet Products in Type 1 Diabetes–Chapter 2: Source Pigs,” Xenotransplantation 16 (2009): 215–222, 10.1111/j.1399-3089.2009.00541.x. 19799761

[5] J. Noordergraaf , A. Schucker , M. Martin , et al., “Pathogen Elimination and Prevention Within a Regulated, Designated Pathogen Free, Closed Pig Herd for Long‐Term Breeding and Production of Xenotransplantation Materials,” Xenotransplantation 25 (2018): e12428, 10.1111/xen.12428. 30264879 PMC7169735

[6] K. Lopata , E. Wojdas , R. Nowak , P. Lopata , and U. Mazurek , “Porcine Endogenous Retrovirus (PERV)‐Molecular Structure and Replication Strategy in the Context of Retroviral Infection Risk of Human Cells,” Frontiers in Microbiology 9 (2018): 1–11, 10.3389/fmicb.2018.00730. 29755422 PMC5932395

[7] J. Denner and R. R. Tönjes , “Infection Barriers to Successful Xenotransplantation Focusing on Porcine Endogenous Retroviruses,” Clinical Microbiology Reviews 25 (2012): 318–343, 10.1128/CMR.05011-11. 22491774 PMC3346299

[8] S. Halecker , L. Krabben , Y. Kristiansen , et al., “Rare Isolation of Human‐Tropic Recombinant Porcine Endogenous Retroviruses PERV‐A/C From Göttingen Minipigs,” Virology Journal 19 (2022): 30, 10.1186/s12985-022-01742-0. 35189916 PMC8862210

[9] S. I. Martin , R. Wilkinson , and J. A. Fishman , “Genomic Presence of Recombinant Porcine Endogenous Retrovirus in Transmitting Miniature Swine,” Virology Journal 3 (2006): 91, 10.1186/1743-422X-3-91. 17081300 PMC1635704

[10] N. Pal , R. Baker , S. Schalk , et al., “Detection of Porcine Endogenous Retrovirus (PERV) Viremia in Diseased versus Healthy US Pigs by Qualitative and Quantitative Real‐Time RT‐PCR,” Transboundary and Emerging Diseases 58 (2011): 344–351, 10.1111/j.1865-1682.2011.01210.x. 21396084

[11] J. Denner , “Recombinant Porcine Endogenous Retroviruses (PERV‐A/C): A New Risk for Xenotransplantation?,” Archives of Virology 153 (2008): 1421–1426, 10.1007/s00705-008-0141-7. 18584115

[12] B. Bartosch , D. Stefanidis , R. Myers , et al., “Evidence and Consequence of Porcine Endogenous Retrovirus Recombination,” Journal of Virology 78 (2004): 13880–13890, 10.1128/JVI.78.24.13880-13890.2004. 15564496 PMC533951

[13] J. Denner , “Risk of Pathogenic Virus Transmission by Somatic Cell Nuclear Transfer: Implications for Xenotransplantation,” Biology of Reproduction 107 (2022): 717–722, 10.1093/biolre/ioac120. 35699429

[14] J. Denner , “How Active Are Porcine Endogenous Retroviruses (PERVs)?,” Viruses 8 (2016): 215, 10.3390/v8080215. 27527207 PMC4997577

[15] J. Denner , R. R. Tönjes , Y. Takeuchi , J. Fishman , and L. Scobie , “First Update of the International Xenotransplantation Association Consensus Statement on Conditions for Undertaking Clinical Trials of Porcine Islet Products in Type 1 Diabetes—Chapter 5: Recipient Monitoring and Response Plan for Preventing Disease Transmission,” Xenotransplantation 23 (2016): 53–59. 26918415 10.1111/xen.12227

[16] J. Denner , H.‐J. Schuurman , and C. Patience , “The International Xenotransplantation Association Consensus Statement on Conditions for Undertaking Clinical Trials of Porcine Islet Products in Type 1 Diabetes–Chapter 5: Strategies to Prevent Transmission of Porcine Endogenous Retroviruses,” Xenotransplantation 16 (2009): 239–248, 10.1111/j.1399-3089.2009.00544.x. 19799764

[17] Y. Luhan , G. Marc , N. Dong , et al., “Genome‐Wide Inactivation of Porcine Endogenous Retroviruses (PERVs),” Science (New York, New York) 350 (1979): 1101–1104. 10.1126/science.aad1191 26456528

[18] D. Niu , H.‐J. Wei , L. Lin , et al., “Inactivation of Porcine Endogenous Retrovirus in Pigs Using CRISPR‐Cas9,” Science (New York, New York) 357 (2017): 1303–1307, 10.1126/science.aan4187. PMC5813284 28798043

[19] J. Cao , Q. Xiao , and Q. Yan , “The Multiplexed CRISPR Targeting Platforms,” Drug Discovery Today: Technologies 28 (2018): 53–61, 10.1016/j.ddtec.2018.01.001. 30205881 PMC6699180

[20] U. Fiebig , K. Fischer , A. Bähr , et al., “Porcine Endogenous Retroviruses: Quantification of the Copy Number in Cell Lines, Pig Breeds, and Organs,” Xenotransplantation 25 (2018): e12445, 10.1111/xen.12445. 30264881

[21] M. A. M. Groenen , A. L. Archibald , H. Uenishi , et al., “Analyses of Pig Genomes Provide Insight Into Porcine Demography and Evolution,” Nature 491 (2012): 393–398, 10.1038/nature11622. 23151582 PMC3566564

[22] A. Warr , N. Affara , B. Aken , et al., “An Improved Pig Reference Genome Sequence to Enable Pig Genetics and Genomics Research,” Gigascience 9 (2020): giaa051, 10.1093/gigascience/giaa051. 32543654 PMC7448572

[23] J.‐Q. Chen , M.‐P. Zhang , X.‐K. Tong , et al., “Scan of the Endogenous Retrovirus Sequences Across the Swine Genome and Survey of Their Copy Number Variation and Sequence Diversity Among Various Chinese and Western Pig Breeds,” Zoological Research 43 (2022): 423, 10.24272/j.issn.2095-8137.2021.379. 35437972 PMC9113972

[24] K. Kono , K. Kataoka , Y. Yuan , et al., “Infectivity Assessment of Porcine Endogenous Retrovirus Using High‐Throughput Sequencing Technologies,” Biologicals 71 (2021): 1–8, 10.1016/j.biologicals.2021.05.001. 34039532

[25] J. Wu , Y. Ma , M. Lv , et al., “Large‐Scale Survey of Porcine Endogenous Retrovirus in Chinese Miniature Pigs,” Comparative Immunology, Microbiology & Infectious Diseases 31 (2008): 367–371, 10.1016/j.cimid.2007.06.004. 17689611

[26] Y. Takeuchi , C. Patience , S. Magre , et al., “Host Range and Interference Studies of Three Classes of Pig Endogenous Retrovirus,” Journal of Virology 72 (1998): 9986–9991, 10.1128/JVI.72.12.9986-9991.1998. 9811736 PMC110514

[27] B. Dieckhoff , B. Kessler , D. Jobst , et al., “Distribution and Expression of Porcine Endogenous Retroviruses in Multi‐Transgenic Pigs Generated for Xenotransplantation,” Xenotransplantation 16 (2009): 64–73, 10.1111/j.1399-3089.2009.00515.x. 19392721

[28] D. Kaulitz , D. Mihica , C. Adlhoch , M. Semaan , and J. Denner , “Improved Pig Donor Screening Including Newly Identified Variants of Porcine Endogenous Retrovirus‐C (PERV‐C),” Archives of Virology 158 (2013): 341–348, 10.1007/s00705-012-1490-9. 23053520

[29] S. Swart , A. Robinson , D. Jonouchi , et al., Metabolic Characterization of Porcine Hepatocytes (Northwestern College, 2024).

[30] Y. Martina , S. Kurian , S. Cherqui , et al., “Pseudotyping of Porcine Endogenous Retrovirus by Xenotropic Murine Leukemia Virus in a Pig Islet Xenotransplantation Model,” American Journal of Transplantation 5 (2005): 1837–1847, 10.1111/j.1600-6143.2005.00978.x. 15996230

[31] D. Kaulitz , D. Mihica , J. Dorna , et al., “Development of Sensitive Methods for Detection of Porcine Endogenous Retrovirus‐C (PERV‐C) in the Genome of Pigs,” Journal of Virological Methods 175 (2011): 60–65, 10.1016/j.jviromet.2011.04.017. 21539860

[32] H. Jhelum , D. Kunec , V. Papatsiros , B. B. Kaufer , and J. Denner , “Reliable Polymerase Chain Reaction Methods for Screening for Porcine Endogenous Retroviruses‐C (PERV‐C) in Pigs,” Viruses 17 (2025): 164, 10.3390/v17020164. 40006919 PMC11860680

[33] Y. Ishida , K. Zhao , A. D. Greenwood , and A. L. Roca , “Proliferation of Endogenous Retroviruses in the Early Stages of a Host Germ Line Invasion,” Molecular Biology and Evolution 32 (2015): 109–120, 10.1093/molbev/msu275. 25261407 PMC4271524

[34] M. Hanke and M. Wink , “Direct DNA Sequencing of PCR‐Amplified Vector Inserts Following Enzymatic Degradation of Primer and dNTPs,” Biotechniques 17 (1994): 858–860. 7639844

[35] Y. Ishida , Y. Demeke , P. J. van Coeverden de Groot , et al., “Distinguishing Forest and Savanna African Elephants Using Short Nuclear DNA Sequences,” Journal of Heredity 102 (2011): 610–616, 10.1093/jhered/esr073. 21775678

[36] M. Mahmoud , Y. Huang , K. Garimella , et al., “Utility of Long‐Read Sequencing for All of Us,” Nature Communications 15 (2024): 837, 10.1038/s41467-024-44804-3. PMC10822842 38281971

[37] K. Katoh and D. M. Standley , “MAFFT Multiple Sequence Alignment Software Version 7: Improvements in Performance and Usability,” Molecular Biology and Evolution 30 (2013): 772–780, 10.1093/molbev/mst010. 23329690 PMC3603318

[38] F. Madeira , N. Madhusoodanan , J. Lee , et al., “The EMBL‐EBI Job Dispatcher Sequence Analysis Tools Framework in 2024,” Nucleic Acids Research 52 (2024): W521–W525, 10.1093/nar/gkae241. 38597606 PMC11223882

[39] S. F. Altschul , W. Gish , W. Miller , E. W. Myers , and D. J. Lipman , “Basic Local Alignment Search Tool,” Journal of Molecular Biology 215 (1990): 403–410, 10.1016/S0022-2836(05)80360-2. 2231712

[40] H. Cheng , G. T. Concepcion , X. Feng , H. Zhang , and H. Li , “Haplotype‐Resolved De Novo Assembly Using Phased Assembly Graphs With Hifiasm,” Nature Methods 18 (2021): 170–175, 10.1038/s41592-020-01056-5. 33526886 PMC7961889

[41] G. Madrigal , B. F. Minhas , and J. Catchen , “Klumpy: A Tool to Evaluate the Integrity of Long‐Read Genome Assemblies and Illusive Sequence Motifs,” Molecular Ecology Resources 25 (2025): e13982, 10.1111/1755-0998.13982. 38800997 PMC11646305

[42] H. Li , “Minimap2: Pairwise Alignment for Nucleotide Sequences,” Bioinformatics 34 (2018): 3094–3100, 10.1093/bioinformatics/bty191. 29750242 PMC6137996

[43] W. Y. Jung , J. E. Kim , K. C. Jung , et al., “Comparison of PERV Genomic Locations Between Asian and European Pigs,” Animal Genetics 41 (2010): 89–92, 10.1111/j.1365-2052.2009.01953.x. 19781037

[44] H. Du , S. Lu , Q. Huang , L. Zhou , and J.‐F. Liu , “Chromosome‐Level Genome Assembly of Huai Pig (Sus scrofa),” Scientific Data 11 (2024): 1072, 10.1038/s41597-024-03921-w. 39358406 PMC11446922

[45] R. Zhou , S. Li , W. Yao , et al., “The Meishan Pig Genome Reveals Structural Variation‐Mediated Gene Expression and Phenotypic Divergence Underlying Asian Pig Domestication,” Molecular Ecology Resources 21 (2021): 2077–2092, 10.1111/1755-0998.13396. 33825319

[46] H. J. Moon , H. K. Kim , S. J. Park , et al., “Comparison of the Age‐Related Porcine Endogenous Retrovirus (PERV) Expression Using Duplex RT‐PCR,” Journal of Veterinary Science 10 (2009): 317–322, 10.4142/jvs.2009.10.4.317. 19934597 PMC2807268

[47] U. Fiebig , L. Krüger , and J. Denner , “Determination of the Copy Number of Porcine Endogenous Retroviruses (PERV) in Auckland Island Pigs Repeatedly Used for Clinical Xenotransplantation and Elimination of PERV‐C,” Microorganisms 12 (2024): 98, 10.3390/microorganisms12010098. 38257925 PMC10820294

