# Supplementary material for: Genomic Validation of PERV‐C‐Free Pigs to Support Xenotransplantation
Source: Xenotransplantation. 2026 Jan 16;33(1):e70109. doi: 10.1111/xen.70109 (PMC12810672; doi:10.1111/xen.70109)
Supplement: Supplementary file 1 — Supporting File 1: xen70109‐sup‐0001‐SupMat.docx [file XEN-33-e70109-s001.docx]

Additional Supplementary figures for Genomic validation of PERV-C-free pigs to support xenotransplantation

| CLUSTAL format alignment by MAFFT FFT-NS-2 (v7.310)  PERV_A_ENV_cons atgcatcccacgttaagccggcgccacctcccgattcggggtggaaagccgaaaagactg  PERV_B_ENV_cons atgcatcccacgttaagctggcgccacctcccgactcggggtggagagccgaaaagactg  PERV_C_ENV_cons atgcatcccacgttaagccggcgccacctcccgattcggggtggaaagccgaaaagactg  ******************.***************.**********.**************  PERV_A_ENV_cons aaaatccccttaagcttcgcctccatcgcgtggttccttactctgtcaataactcctcaa  PERV_B_ENV_cons agaatccccttaagcttcgcctccatcgcctggttccttactctaacaataactccccag  PERV_C_ENV_cons aaaatccccttaagcttcgcctccatcgcgtggttccttactctgtcaataacctctcag  *.*************************** **************. *******..*.**.  PERV_A_ENV_cons gttaatggtaaacgccttgtggacagcccgaactcccataaacccttatctctcacctgg  PERV_B_ENV_cons gccagtagtaaacgccttatagacagctcgaacccccatagacctttatcccttacctgg  PERV_C_ENV_cons actaatggtatgcgcataggagacagcctgaactcccataaacccttatctctcacctgg  ...*.*.*** .*** * . .******..****.******.***.*****.**.******  PERV_A_ENV_cons ttacttactgactccggtacaggtattaatattaacagcactcaaggggaggctcccttg  PERV_B_ENV_cons ctgattattgaccctgatacgggtgtcactgtaaatagcactcgaggtgttgctcctaga  PERV_C_ENV_cons ttaattactgactccggcacaggtattaatatcaacaacactcaaggggaggctccttta  .*. ***.****.*.*..**.***.*.* *.* **.*.*****.*** * *****. .  PERV_A_ENV_cons gggacctggtggcctgaattatatgtctgccttcgatcagtaatccctggtctcaatgac  PERV_B_ENV_cons ggcacctggtggcctgaactgcatttctgcctccgattgattaaccccgctgttaa----  PERV_C_ENV_cons ggaacctggtggcctgatctatacgtttgcctcagatcagttattcctagtct-------  ** ************** .*..*. *.*****. ***...* * .**.. * *  PERV_A_ENV_cons caggccacaccccccgatgtactccgtgcttacgggttttacgtttgcccaggaccccca  PERV_B_ENV_cons --aagcacacctcccaacctagtccgtagttatgggttctattgctgcccagg---caca  PERV_C_ENV_cons --gacctcacccccagatatcctccatgctcacggattttatgtttgcccaggaccacca  .. * ****.** .*. * ***.*. *.*.**.**.**. .******** **  PERV_A_ENV_cons aataatgaagaatattgtggaaatcctcaggatttcttttgcaagcaatggagctgcgta  PERV_B_ENV_cons gagaaagagaaatactgtgggggttctggggaatccttctgtaggagatggagctgcgtc  PERV_C_ENV_cons aataatggaaaacattgcggaaatcccagagatttcttttgtaaacaatggaactgtgta  .* ** *...**.*.**.**...*.*. ..** *.***.**.*.. .*****.***.**  PERV_A_ENV_cons acttctaatgatgggaattggaaatggccagtctctcagcaagacagagtaagttactct  PERV_B_ENV_cons acctccaacgatggagactggaaatggccgatctctctccaggaccgggtaaaattctcc  PERV_C_ENV_cons acctctaatgatggatattggaaatggccaacctctcagcaggatagggtaagtttttct  **.**.**.*****. *.***********...***** **.**. *.****. * .**.  PERV_A_ENV_cons tttgttaacaatcctaccagttataatcaatttaatta--tggccatgggagatggaaag  PERV_B_ENV_cons tttgtcaattc-----------------------------cggcc---------------  PERV_C_ENV_cons tatgtcaacacctataccagctctggacaatttaattacctgacc------------tgg  * ***.**. .*.**  PERV_A_ENV_cons attggcaacagcgggtacaaaaagatg-tacgaaataagcaaataagctgtcattcgtta  PERV_B_ENV_cons -------cgggcaagtacaaagtgatgaaactatataaagataagagctgctccccatca  PERV_C_ENV_cons attagaactggaagccccaag---------------------------tgctctccttca  .* .. . ***. **.. ..* *.*  PERV_A_ENV_cons gacctagattacttaaaaataagtttcactgaaaaaggaaaacaagaaaatattcaaaag  PERV_B_ENV_cons gacttagattatctaaagataagtttcactgaaaaaggaaaacaggaaaatattcaaaag  PERV_C_ENV_cons gacctagattacctaaaaataagtttcactgagaaaggaaaacaagaaaatatcctaaaa  ***.*******..****.**************.***********.********.* ***.  PERV_A_ENV_cons tgggtaaatggtatgtcttggggaatagtgtactatggaggctctgggagaaagaaagga  PERV_B_ENV_cons tggataaatggtatgagctggggaatagttttttataaa---tatggcgggggagcaggg  PERV_C_ENV_cons tgggtaaatggtatgtcttggggaatggtatattatggaggctcgggtaaacaaccaggc  ***.*********** .********.** * .***..* * ** ... .. ***  PERV_A_ENV_cons tctgttctgactattcgcctcagaatagaaactcagatggaacctccggttgctatagga  PERV_B_ENV_cons tccactttaaccattcgccttaggatagagacggggacagaaccccctgtggcagtggga  PERV_C_ENV_cons tccattctaactattcgcctcaaaataaa---ccagctggagcctccaatggctatagga  **...*.*.**.********.*..***.* .* ..**.**.** .* ** .*.***  PERV_A_ENV_cons ccaaataagggtttggccgaacaaggacctccaatccaagaacagaggccatctcctaac  PERV_B_ENV_cons cccgataaagtactggctgaacaggggcccccggcc------ctggagccaccgcataac  PERV_C_ENV_cons ccaaatacggtcttgacgggtcaaagacccccaacccaaggaccaggaccatcctctaac  ** .*** .* .**.* *. **..*.**.**...* * ....***.* . ****  PERV_A_ENV_cons cc-------ctctgatta-----------------------------------caataca  PERV_B_ENV_cons ttgccggtgccccaattaacctcgctgcggcctgacataacacagccgcctagcaacggt  PERV_C_ENV_cons ---------------------------------------------------------ata  .  PERV_A_ENV_cons acctctggatcagtccccactga---------------gcctaacatcactattaaaaca  PERV_B_ENV_cons accactggattgattcctaccaacacgcctagaaactccccaggtgttcctgttaagaca  PERV_C_ENV_cons acttctggatcagaccccactga---------------gtctaacagcacgactaaaatg  **. ******... .**.**..* .* .... . * ..***.*..  PERV_A_ENV_cons ggggcgaaactttttagcctcatccagggagcttttcaagctcttaactccacgactcca  PERV_B_ENV_cons ggacagagactcttcagtctcatccagggagctttccaagccatcaactccaccgaccct  PERV_C_ENV_cons ggggcaaaactttttagcctcatccagggagcttttcaagctcttaactccacgactcca  **. .*.***.**.**.*****************.*****. *.******** . .**  PERV_A_ENV_cons gaggctacctcttcttgttggctttgcttagcttcgggcccaccttactatgagggaatg  PERV_B_ENV_cons gatgccacttcttcttgttggctttgtctatcctcagggcctccttattatgaggggatg  PERV_C_ENV_cons gaggctacctcttcttgttggctatgcttagctttgggcccaccttactatgaaggaatg  ** **.**.************** **..** *.*..** ** *****.*****.**.***  PERV_A_ENV_cons gctagaggagggaaattcaatgtgacaaaggaacatagagaccaatgtacatggggatcc  PERV_B_ENV_cons gctaaagaaggaaaattcaatgtgaccaaagagcatagaaatcaatgtacatgggggtcc  PERV_C_ENV_cons gctagaagagggaaattcaatgtgacaaaagaacatagagaccaatgcacatggggatcc  ****.*..***.************** **.**.******.*.*****.********.***  PERV_A_ENV_cons caaaataagcttacccttactgaggtttctggaaaaggcacctgcatagggatggttccc  PERV_B_ENV_cons cgaaataagcttaccctcactgaagtttccgggaaggggacatgcataggaaaagctccc  PERV_C_ENV_cons caaaataagcttacccttactgaggtttctggaaaaggcacctgcataggaaaggttccc  *.***************.*****.*****.**.**.** ** ********.* .*.****  PERV_A_ENV_cons ccatcccaccaacacctttgtaaccacactgaagcctttaatcgaacctctgagagtcna  PERV_B_ENV_cons ccatcccaccaacacctttgctatagtactgtggtttatgagcaggcctcagaaaatc-a  PERV_C_ENV_cons ccatcccaccaacacctttgtaaccacactgaagcctttaatcaaacctctgagagtc-a  ********************. *. ..**** .*..* *.* *...**** **.*.** *  PERV_A_ENV_cons gtatctggtacctggttatgacaggtggtgggcatgtaatactggattaaccccttgtgt  PERV_B_ENV_cons gtatttagtacctggttataacaggtggtgggcatgcaatactgggttaaccccctgtgt  PERV_C_ENV_cons atatctggtacctggttatgacaggtggtgggcatgtaatactggattaaccccttgtgt  .***.*.************.****************.********.********.*****  PERV_A_ENV_cons ttccaccttggttttcaaccaaactaaagacttttgcgttatggtccaaattgtcccccg  PERV_B_ENV_cons ttccacctcagtcttcaaccaatccaaagatttctgtgtcatggtccaaatcgtcccccg  PERV_C_ENV_cons ttccactttggtttttaaccaaactaaagatttttgcattatggtccaaattgttccccg  ******.*..**.**.****** *.*****.**.**..*.***********.**.*****  PERV_A_ENV_cons ggtgtactactatcccgaaaaagcagtccttgatgaatatgactatagatataatcggcc  PERV_B_ENV_cons agtgtactaccatcctgaggaagtggtccttgatgaatatgactatcggtataaccgacc  PERV_C_ENV_cons agtgtattactatcccgaaaaagcaatccttgatgaatatgactacagaaatcatcgaca  .*****.***.****.**..***...*******************. *. ** *.**.*  PERV_A_ENV_cons aaaaagagagcccatatccctgacactagctgtaatgctcggattgggagtggctgcagg  PERV_B_ENV_cons aaaaagagaacccgtatcccttaccctagctgtaatgctcggattagggacggccgttgg  PERV_C_ENV_cons aaagagagaacccatatctctgacacttgctgtgatgctcggacttggagtggcagcagg  ***.*****.***.****.** ** ** *****.*********.* **...*** *. **  PERV_A_ENV_cons cgtgggaacaggaacggctgccctaatcacaggaccgcaacagctggagaaaggacttag  PERV_B_ENV_cons cgtaggaacagggacagctgccctgatcacaggaccacagcagctagagaaaggacttgg  PERV_C_ENV_cons tgtaggaacaggaacagctgccctggtcacgggaccacagcagctagaaacaggacttag  .**.********.**.********..****.*****.**.*****.**.* *******.*  PERV_A_ENV_cons taacctacatcgaattgtaacggaagatctccaagccctagaaaaatctgtcagtaacct  PERV_B_ENV_cons tgagctacatgcggccatgacagaagatctccgagccttagaggagtctgttagcaacct  PERV_C_ENV_cons taacctacatcgaattgtaacagaagatctccaagccctagaaaaatctgtcagtaacct  *.* ****** .....*.**.**********.****.****..*.*****.**.*****  PERV_A_ENV_cons ggaggaatccctaacctccttatctgaagtggttctacagaacagaagggggttagatct  PERV_B_ENV_cons agaagagtccctgacttctttgtctgaagtggttctacagaaccggaggggattagatct  PERV_C_ENV_cons ggaggaatccctaacctccttatctgaagtagtcctacagaatagaagagggttagattt  .**.**.*****.**.**.**.********.**.********. *.**.**.******.*  PERV_A_ENV_cons gttatttctaaaagaaggagggttatgtgtagccttaaaagaggaatgctgcttctatgt  PERV_B_ENV_cons gctgtttctaagagaaggtgggttatgtgcagccttaaaagaagaatgttgcttctatgt  PERV_C_ENV_cons attatttctaaaagaaggaggattatgtgtagccttgaaggaggaatgctgtttttatgt  ..*.*******.****** **.*******.******.**.**.*****.**.**.*****  PERV_A_ENV_cons agatcactcaggagccatcagagactccatgagcaagcttagagaaaggttagagaggcg  PERV_B_ENV_cons agatcactcaggagccatcagagactccatgagcaagcttagagaaaggttagagaggcg  PERV_C_ENV_cons ggatcattcaggggccatcagagactccatgaacaagcttagagaaaggttggagaagcg  .*****.*****.*******************.******************.****.***  PERV_A_ENV_cons tcgaagggaaagagaggctgaccaggggtggtttgaaggatggttcaacaggtctccttg  PERV_B_ENV_cons tcgaagggaaagagaggctgaccaggggtggtttgaaggatggttcaacaggtctccttg  PERV_C_ENV_cons tcgaagggaaaaggaaactactcaagggtggtttgagggatggttcaacaggtctccttg  ***********..**..**. .**.***********.***********************  PERV_A_ENV_cons gatgaccaccctgctttctgctctgacgggacccctagtagtcctgctcctgttacttac  PERV_B_ENV_cons gatgaccaccctgctttctgctctgacgggacccctagtagtcctgctcctgttacttac  PERV_C_ENV_cons gttggctaccctactttctgctttaacaggacccttaatagtcctcctcctgttactcac  * **.*.*****.*********.*.**.******.**.******* ***********.**  PERV_A_ENV_cons agttgggccttgcttaattaataggtttgttgcctttgttagagaacgagtgagtgcagt  PERV_B_ENV_cons agttgggccttgcttaattaataggtttgttgcctttgttagagaacgagtgagtgcagt  PERV_C_ENV_cons agttgggccatgtattattaacaagttaattgccttcattagagaacgaataagtgcagt  ********* **. * *****.*.*** .*******..***********.*.********  PERV_A_ENV_cons ccagatcatggtacttaggcaacagtaccaaggccttctgagccaa--------------  PERV_B_ENV_cons ccagatcatggtacttaggcaacagtaccaaggccttctgagccaaggagaaactgacct  PERV_C_ENV_cons ccagatcatggtacttagacaacagtaccaaagcccgtctagcaggg---aagctggccg  ******************.************.***. .. *** ..  PERV_A_ENV_cons ----  PERV_B_ENV_cons ctag  PERV_C_ENV_cons ctag |
| --- |

Supplementary Figure 2: Mafft v7.10 sequence alignment of all three consensus sequences for the envelope regions for PERVs A, B, and C. Highlighted is a 50 basepair region in the PERV-C consensus sequence that was found shared across all PERV-C accession sequences and is unique to PERV-C.

| CLUSTAL format alignment by MAFFT FFT-NS-2 (v7.310)  HQ536013.1 atgcatcccacgttaagccggcgccacctcccgattcggggtggaaagccgaaaagactg  HQ536015.1 atgcatcccacgttaagccggcgccacctcccgattcggggtggaaagccgaaaagactg  HQ536016.1 atgcatcccacgttaagccggcgccacctcccgattcggggtggaaagccgaaaagactg  AF038600.1 atgcatcccacgttaaaccggcgccacctcccgattcggggtggaaagccgaaaagactg  ****************.*******************************************  HQ536013.1 aaaatccccttaagcttcgcctccatcgcgtggttccttactctgtcaataacctctcag  HQ536015.1 aaaatccccttaagcttcgcctccatcgcgtggttccttactctgtcaataacctctcag  HQ536016.1 aaaatccccttaagcttcgcctccatcgcgtggttccttactctgtcaataacctctcag  AF038600.1 aaaatccccttaagcttcgcctccatcgcgtggttccttactctgtcaataacctctcag  ************************************************************  HQ536013.1 actaatggtatgcgcataggagacagcctgaactcccataaacccttatctctcacctgg  HQ536015.1 actaatggtatgcgcataggagacagcctgaactcccataaacccttatctctcacctgg  HQ536016.1 actaatggtatgcgcataggagacagcctgaactcccataaacccttatctctcacctgg  AF038600.1 actaatggtatgcgcataggagacagcctgaactcccataaacccttatctctcacctgg  ************************************************************  HQ536013.1 ttaattactgactccggcacaggtattaatatcaacaacactcaaggggaggctccttta  HQ536015.1 ttaattactgactccggcacaggtattaatatcaacaacactcaaggggaggctccttta  HQ536016.1 ttaattactgactccggcacaggtattaatatcaacaacactcaaggggaggctccttta  AF038600.1 ttaattactgactccggcacaggtattaatatcaacaacactcaaggggaggctccttta  ************************************************************  HQ536013.1 ggaacctggtggcctgatctatacgtttgcctcagatcagttattcctagtctgacctca  HQ536015.1 ggaacctggtggcctgatctatacgtttgcctcagatcagttattcctagtctgacctca  HQ536016.1 ggaacctggtggcctgatctatacgtttgcctcagatcagttattcctagtctgacctca  AF038600.1 ggaacctggtggcctgatctatacgtttgcctcagatcagttattcctagtctgacctca  ************************************************************  HQ536013.1 cccccagatatcctccgtgctcacggattttatgtttgcccaggaccaccaaataatgga  HQ536015.1 cccccagatatcctccatgctcacggattttatgtttgcccaggaccaccaaataatgga  HQ536016.1 cccccagatatcctccatgctcacggattttatgtttgcccaggaccaccaaataatgga  AF038600.1 cccccagatatcctccatgctcacggattttatgtttgcccaggaccaccaaataatgga  ****************.*******************************************  HQ536013.1 aaacattgcggaaatcccagagatttcttttgtaaacaatggaactgtgtaacctctaat  HQ536015.1 aaacattgcggaaatcccagagatttcttttgtaaacaatggaactgtgtaacctctaat  HQ536016.1 aaacattgcggaaatcccagagatttcttttgtaaacaatggaactgtgtaacctctaat  AF038600.1 aaacattgcggaaatcccagagatttcttttgtaaacaatggaactgtgtaacctctaat  ************************************************************  HQ536013.1 gatggatattggaaatggccaacctctcagcaggatagggtaagtttttcttatgtcaac  HQ536015.1 gatggatattggaaatggccaacctctcagcaggatagggtaagtttttcttatgtcaac  HQ536016.1 gatggatattggaaatggccaacctctcagcaggatagggtaagtttttcttatgtcaac  AF038600.1 gatggatattggaaatggccaacctctcagcaggatagggtaagtttttcttatgtcaac  ************************************************************  HQ536013.1 acctataccagctctggacaatttaattacctgacctggattagaactggaagccccaag  HQ536015.1 acctataccagctctgaacaatttaattacctgacctggattagaactggaagccccaag  HQ536016.1 acctataccagctctggacaatttaattacctgacctggattagaactggaagccccaag  AF038600.1 acctataccagctctggacaatttaattacctgacctggattagaactggaagccccaag  ****************.*******************************************  HQ536013.1 tgctctccttcagacctagattacctaaaaataagtttcactgagaaaggaaaacaagaa  HQ536015.1 tgctctccttcagacctagattacctaaaaataagtttcactgagaaaggaaaacaagaa  HQ536016.1 tgctctccttcagacctagattacctaaaaataagtttcactgagaaaggaaaacaagaa  AF038600.1 tgctctccttcagacctagattacctaaaaataagtttcactgagaaaggaaaacaagaa  ************************************************************  HQ536013.1 aatatcctaaaatgggtaaatggtatgtcttggggaatggtatattatggaggctcgggt  HQ536015.1 aatatcctaaaatgggtaaatggtatgtcttggggaatggtatattatggaggctcgggt  HQ536016.1 aatatcctaaaatgggtaaatggtatgtcttggggaatggtatattatggaggctcgggt  AF038600.1 aatatcctaaaatgggtaaatggtatgtcttggggaatggtatattatggaggctcgggt  ************************************************************  HQ536013.1 aaacaaccaggctccattctaactattcgcctcaaaataaaccagctggagcctccaatg  HQ536015.1 aaacaaccaggctccattctaactattcgcctcaaaataaaccagctggagcctccaatg  HQ536016.1 aaacaaccaggctccattctaactattcgcctcaaaataaaccagctggagcctccaatg  AF038600.1 aaacaaccaggctccattctaactattcgcctcaaaataaaccagctggagcctccaatg  ************************************************************  HQ536013.1 gctataggaccaaatacggtcttgacgggtcaaagacccccaacccaaggaccaggacca  HQ536015.1 gctataggaccaaatacggtcttgacgggtcaaagacccccaacccaaggaccaggacca  HQ536016.1 gctataggaccaaatacggtcttgacgggtcaaagacccccaacccaaggaccaggacca  AF038600.1 gctataggaccaaatacggtcttgacgggtcaaagacccccaacccaaggaccaggacca  ************************************************************  HQ536013.1 tcctctaacataacttctggatcagaccccactgagtctaacagcacgactaaaatgggg  HQ536015.1 tcctctaacataacttctggatcagaccccactgagtctaacagcacgactaaaatgggg  HQ536016.1 tcctctaacataacttctggatcagaccccactgagtctaacagcacgactaaaatgggg  AF038600.1 tcctctaacataacttctggatcagaccccactgagtctagcagcacgactaaaatgggg  ****************************************.*******************  HQ536013.1 gcaaaactttttagcctcatccagggagcttttcaagctcttaactccacgactccagag  HQ536015.1 gcaaaactttttagcctcatccagggagcttttcaagctcttaactccacgactccagag  HQ536016.1 gcaaaactttttagcctcatccagggagcttttcaagctcttaactccacgactccagag  AF038600.1 gcaaaactttttagcctcatccagggagcttttcaagctcttaactccacgactccagag  ************************************************************  HQ536013.1 gctacctcttcttgttggctatgcttagctttgggcccaccttactatgaaggaatggct  HQ536015.1 gctacctcttcttgttggctttgcttagcttcgggcccaccttactatgagggaatggct  HQ536016.1 gctacctcttcttgttggctatgcttagctttgggcccaccttactatgaaggaatggct  AF038600.1 gctacctcttcttgttggctatgcttagcttcgggcccaccttactatgaaggaatggct  ******************** **********.******************.*********  HQ536013.1 agaagagggaaattcaatgtgacaaaagaacatagagaccaatgcacatggggatcccaa  HQ536015.1 agaagagggaaattcaatgtgacaaaagaacatagagaccaatgcacatggggatcccaa  HQ536016.1 agaagagggaaattcaatgtgacaaaagaacatagagaccaatgcacatggggatcccaa  AF038600.1 agaagagggaaattcaatgtgacaaaagaacatagagaccaatgcacatggggatcccaa  ************************************************************  HQ536013.1 aataagcttacccttactgaggtttctggaaaaggcacctgcataggaaaggttccccca  HQ536015.1 aataagcttacccttactgaggtttctggaaaaggcacctgcataggaaaggttccccca  HQ536016.1 aataagcttacccttactgaggtttctggaaaaggcacctgcataggaaaggttccccca  AF038600.1 aataagcttacccttactgaggtttctggaaaaggcacctgcataggaaaggttccccca  ************************************************************  HQ536013.1 tcccaccaacacctttgtaaccacactgaagcctttaatcaaacctctgagagtcaatat  HQ536015.1 tcccaccaacacctttgtaaccacactgaagcctttaatcaaacctctgagagtcaatat  HQ536016.1 tcccaccaacacctttgtaaccacactgaagcctttaatcaaacctctgagagtcaatat  AF038600.1 tcccaccaacacctttgtaaccacactgaagcctttaatcaaacctctgagagtcaatat  ************************************************************  HQ536013.1 ctggtacctggttatgacaggtggtgggcatgtaatactggattaaccccttgtgtttcc  HQ536015.1 ctggtacctggttatgacaggtggtgggcatgtaatactggattaaccccttgtgtttcc  HQ536016.1 ctggtacctggttatgacaggtggtgggcatgtaatactggattaaccccttgtgtttcc  AF038600.1 ctggtacctggttatgacaggtggtgggcatgtaatactggattaaccccttgtgtttcc  ************************************************************  HQ536013.1 actttggtttttaaccaaactaaagatttttgcattatggtccaaattgttccccgagtg  HQ536015.1 actttggtttttaaccaaactaaagatttttgcattatggtccaaattgttccccgagtg  HQ536016.1 accttggtttttaaccaaactaaagatttttgcattatggtccaaattgttccccgagtg  AF038600.1 accttggtttttaaccaaactaaagatttttgcattatggtccaaattgttccccgagtg  **.*********************************************************  HQ536013.1 tattactatcccgaaaaagcaatccttgatgaatatgactacagaaatcatcgacaaaag  HQ536015.1 tattactatcccgaaaaagcaatccttgatgaatatgactacagaaatcatcgacaaaag  HQ536016.1 tattactatcccgaaaaagcaatccttgatgaatatgactacagaaatcatcgacaaaag  AF038600.1 tattactatcccgaaaaagcaatccttgatgaatatgactacagaaatcatcgacaaaag  ************************************************************  HQ536013.1 agagaacccatatctctgacacttgctgtgatgctcggacttggagtggcagcaggtgta  HQ536015.1 agagaacccatatctctgacacttgctgtgatgctcggacttggagtggcagcaggtgta  HQ536016.1 agagaacccatatctctgacacttgctgtgatgctcggacttggagtggcagcaggtgta  AF038600.1 agagaacccatatctctgacacttgctgtgatgctcggacttggagtggcagcaggtgta  ************************************************************  HQ536013.1 ggaacaggaacagctgccctggtcacgggaccacagcagctagaaacaggacttagtaac  HQ536015.1 ggaacaggaacagctgccctggtcacgggaccacagcagctagaaacaggacttagtaac  HQ536016.1 ggaacaggaacagctgccctggtcacgggaccacagcagctagaaacaggacttagtaac  AF038600.1 ggaacaggaacagctgccctggtcacgggaccacagcagctagaaacaggacttagtaac  ************************************************************  HQ536013.1 ctacatcgaattgtaacagaagatctccaagccctagaaaaatctgtcagtaacctggag  HQ536015.1 ctacatcgaattgtaacagaagatctccaagccctagaaaaatctgtcagtaacctggag  HQ536016.1 ctacatcgaattgtaacagaagatctccaagccctagaaaaatctgtcagtaacctggag  AF038600.1 ctacatcgaattgtaacagaagatctccaagccctagaaaaatctgtcagtaacctggag  ************************************************************  HQ536013.1 gaatccctaacctccttatctgaagtagtcctacagaatagaagagggttagatttatta  HQ536015.1 gaatccctaacctccttatctgaagtagtcctacagaatagaagagggttagatttatta  HQ536016.1 gaatccctaacctccttatctgaagtagtcctacagaatagaagagggttagatttatta  AF038600.1 gaatccctaacctccttatctgaagtagtcctacagaatagaagagggttagatttatta  ************************************************************  HQ536013.1 tttctaaaagaaggaggattatgtgtagccttgaaggaggaatgctgtttttatgtggat  HQ536015.1 tttctaaaagaaggaggattatgtgtagccttgaaggaggaatgctgtttttatgtggat  HQ536016.1 tttctaaaagaaggaggattatgtgtagccttgaaggaggaatgctgtttttatgtggat  AF038600.1 tttctaaaagaaggaggattatgtgtagccttgaaggaggaatgctgtttttatgtggat  ************************************************************  HQ536013.1 cattcaggggccatcagagactccatgaacaagcttagagaaaggttggagaagcgtcga  HQ536015.1 cattcaggggccatcagagactccatgaacaagcttagagaaaggttggagaagcgtcga  HQ536016.1 cattcaggggccatcagagactccatgaacaagcttagagaaaggttggagaagcgtcga  AF038600.1 cattcaggggccatcagagactccatgaacaagcttagagaaaggttggagaagcgtcga  ************************************************************  HQ536013.1 agggaaaaggaaactactcaagggtggtttgagggatggttcaacaggtctccttggttg  HQ536015.1 agggaaaaggaaactactcaagggtggtttgagggatggttcaacaggtctccttggttg  HQ536016.1 agggaaaaggaaactactcaagggtggtttgagggatggttcaacaggtctccttggttg  AF038600.1 agggaaaaggaaactactcaagggtggtttgagggatggttcaacaggtctctttggttg  ****************************************************.*******  HQ536013.1 gctaccctactttctgctttaacaggacccttaatagtcctcctcctgttactcacagtt  HQ536015.1 gctaccctactttctgctttaacaggacccttaatagtcctcctcctgttactcacagtt  HQ536016.1 gctaccctactttctgctttaacaggacccttaatagtcctcctcctgttactcacagtt  AF038600.1 gctaccctactttctgctttaacaggacccttaatagtcctcctcctgttactcacagtt  ************************************************************  HQ536013.1 gggccatgtattattaacaagttaattgccttcattagagaacgaataagtgcagtccag  HQ536015.1 gggccatgtattattaacaagttaattgccttcattagagaacgaataagtgcagtccag  HQ536016.1 gggccatgtattattaataagttaattgccttcattagagaacgaataagtgcagtccag  AF038600.1 gggccatgtattattaacaagttaattgccttcattagagaacgaataagtgcagtccag  *****************.******************************************  HQ536013.1 atcatggtacttagacaacagtaccaaagcccgtctagcagggaagctggccgctag  HQ536015.1 atcatggtacttagacaacagtaccaaagcccgtctagcagggaagctggccgctag  HQ536016.1 atcatggtacttagacaacagtaccaaagcccgtctagcagggaagctggccgctag  AF038600.1 atcatggtacttagacaacagtaccaaagcccgtctagcagggaagctggccgctag  ********************************************************* |
| --- |

Supplementary Figure 3: Mafft v7.10 sequence alignment of all PERV-C accessions used to generate a consensus sequence for PERV-C. Highlighted is a 50 basepair region is shared across all PERV-C accession sequences.


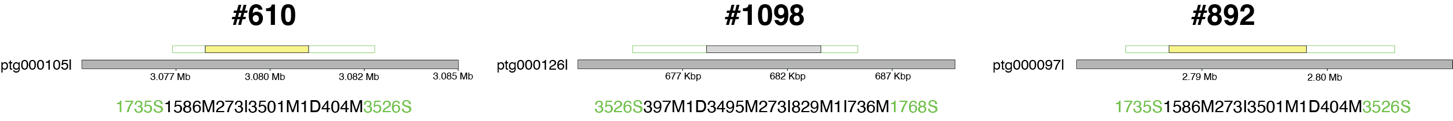


Supplementary Figure 4: Primary alignments of the putative PERV-C locus obtained from #710 onto the genome assemblies of #610, #1098 and #892. The cigar string for each alignment describes a similar case across all three alignments, in which the ends of the alignments are soft clipped (clear green rectangles and green text). Within the aligned region, multiple deletions (letter *D* in the cigar) and insertions (letter *I* in the cigar) detail multiple disagreements with the reference genomes. Yellow alignments for #610 and #892 serve to illustrate alignments onto the reverse strand and the grey alignment for #1098 depicts an alignment on the forward strand.

Supplementary Table 2: Genome assembly statistics for the four pigs sequenced in this study.

|  | Assembly Size | N50 | Number of contigs | Average Coverage |
| --- | --- | --- | --- | --- |
| C710 | 2.796 Gb | 207 Mb | 2,809 | 12.84x |
| C610 | 2.662 Gb | 42 Mb | 1,132 | 16.63x |
| C892 | 2.968 Gb | 287 Mb | 3,221 | 12.47x |
| C1098 | 2.817 Gb | 54 Mb | 1,252 | 17.72x |
