# Supplementary material for: Genomic Validation of PERV‐C‐Free Pigs to Support Xenotransplantation
Source: Xenotransplantation. 2026 Jan 16;33(1):e70109. doi: 10.1111/xen.70109 (PMC12810672; doi:10.1111/xen.70109)
Supplement: Supplementary file 2 — Supporting File 2: xen70109‐sup‐0002‐FigureS1.pdf [file XEN-33-e70109-s002.pdf]

Animal ID	Martina et al.	Sex	Breed	Date of Birth	Results (PCR 1)	Sequencing
C51	Suspect	F	Landrace	23-Sep-19	Positive	PERV-C +
C137	Positive	F	Landrace	23-Sep-19	Positive	PERV-C +
C354	Not tested	F	Landrace/York	31-Jan-21	Positive	PERV-A +
C357	Not tested	F	Landrace/York	31-Jan-21	Positive	PERV-C +
C375	Neg	F	Landrace/York	12-Mar-21	Positive	PERV-A +
C377	Not tested	F	Landrace/York	12-Mar-21	Positive	PERV-A +
C387	Suspect	F	York	24-Mar-21	Positive	PERV-C +
C388	Not tested	F	York	24-Mar-21	Positive	PERV-C +
C404	Positive	F	York	9-Apr-21	Positive	PERV-C +
C417	Positive	F	York	9-Apr-21	Positive	PERV-C +
C418	Suspect	F	York	9-Apr-21	Positive	PERV-C +
C419	Positive	F	York	9-Apr-21	Positive	PERV-C +
C443	Not tested	F	Landrace/York	27-May-21	Positive	PERV-C +
C444	Not tested	F	Landrace/York	27-May-21	Positive	PERV-C +
C446	Positive	M	York	1-Jun-21	Positive	PERV-C +
C448	Positive	M	York	1-Jun-21	Positive	PERV-C +
C453	Positive	F	York	1-Jun-21	Positive	PERV-C +
C454	Suspect	F	York	1-Jun-21	Positive	PERV-C +
C456	Positive	F	York	1-Jun-21	Positive	PERV-C +
C457	Positive	F	York	1-Jun-21	Positive	PERV-C +
C458	Positive	F	York	1-Jun-21	Positive	PERV-C +
C519	Positive	F	Landrace/York	5-Aug-21	Positive	PERV-C +
C543	Negative	F	Landrace/York	7-Sep-21	Positive	PERV-C +
F358-1	Not tested	M	Landrace/York	35d Fetal Fibroblast	Positive	PERV-C +
F358-2	Not tested	F	Landrace/York	35d Fetal Fibroblast	Positive	PERV-C +
F358-3	Not tested	M	Landrace/York	35d Fetal Fibroblast	Positive	PERV-C +
F358-6	Not tested	M	Landrace/York	35d Fetal Fibroblast	Positive	PERV-C +
F358-8	Not tested	M	Landrace/York	35d Fetal Fibroblast	Positive	PERV-A +

Supplementary Figure 1: Results from an initial round of screening of 28 pigs using a single pair of primers (PCR1). All 28 animals tested positive either by gel visualization and/or Sanger sequencing. Among the 17 of 28 animals with prior results based on the primers of Martina et al., 2 had previously been classified by the University of Minnesota Veterinary Diagnostic Lab negative, 4 suspect, and 11 positive.
