# Supplementary material for: Genomic Validation of PERV‐C‐Free Pigs to Support Xenotransplantation
Source: Xenotransplantation. 2026 Jan 16;33(1):e70109. doi: 10.1111/xen.70109 (PMC12810672; doi:10.1111/xen.70109)
Supplement: Supplementary file 3 — Supporting File 3: xen70109‐sup‐0003‐TableS1.pdf [file XEN-33-e70109-s003.pdf]

Date lab received	Breed	Animal ID	Date of Birth	Gender	Assay	Sample type	Result	ct (40 cycle cut off)
10/8/20	Yorkshire	C115	23-Sep-2019	F	Porcine endogenous retrovirus type C PCR	Buffy Coat	Neg	0
1/25/22	Landrace	C137	23-Sep-2019	F	Porcine endogenous retrovirus type C PCR	DNA	POSITIVE	26.43
10/8/20	Yorkshire	C165	23-Sep-2019	M	Porcine endogenous retrovirus type C PCR	Buffy Coat	POSITIVE	30.42
2/1/22	Yorkshire	C20	26-Sep-2019	M	Porcine endogenous retrovirus type C PCR	Serum	Suspect	37.41
2/1/22	Yorkshire	C217	29-Sep-2019	M	Porcine endogenous retrovirus type C PCR	Serum	POSITIVE	35.9
3/30/22	Landrace	C272	2-Oct-2019	F	Porcine endogenous retrovirus type C PCR	Serum	Suspect	37.12
11/5/20	Crossbreed	C298	5-Oct-2019	F	Porcine endogenous retrovirus type C PCR	Blood	Neg	0
11/5/20	Crossbreed	C299	8-Oct-2019	F	Porcine endogenous retrovirus type C PCR	Blood	Neg	0
11/5/20	Crossbreed	C300	11-Oct-2019	F	Porcine endogenous retrovirus type C PCR	Blood	Neg	0
11/5/20	Crossbreed	C301	14-Oct-2019	F	Porcine endogenous retrovirus type C PCR	Blood	Neg	0
11/5/20	Crossbreed	C302	17-Oct-2019	F	Porcine endogenous retrovirus type C PCR	Blood	Neg	0
11/5/20	Crossbreed	C303	20-Oct-2019	F	Porcine endogenous retrovirus type C PCR	Blood	Neg	0
2/1/22	Landrace	C319	23-Oct-2019	M	Porcine endogenous retrovirus type C PCR	Serum	Suspect	36.86
3/30/22	Landrace	C34	26-Oct-2019	F	Porcine endogenous retrovirus type C PCR	Serum	Neg	0
1/25/22	Crossbreed	C375	29-Oct-2019	F	Porcine endogenous retrovirus type C PCR	DNA	Neg	0
3/30/22	Yorkshire	C387	1-Nov-2019	F	Porcine endogenous retrovirus type C PCR	Serum	Suspect	37.94
3/30/22	Yorkshire	C394	4-Nov-2019	F	Porcine endogenous retrovirus type C PCR	Serum	POSITIVE	30.36
1/25/22	Yorkshire	C404	7-Nov-2019	F	Porcine endogenous retrovirus type C PCR	DNA	POSITIVE	27.43
5/20/22	Yorkshire	C406	13-Nov-2019	F	Porcine endogenous retrovirus type C PCR	Serum	Suspect	37.14
3/30/22	Yorkshire	C417	16-Nov-2019	F	Porcine endogenous retrovirus type C PCR	Serum	POSITIVE	33.24
5/20/22	Yorkshire	C418	19-Nov-2019	F	Porcine endogenous retrovirus type C PCR	Serum	Suspect	36.14
1/25/22	Yorkshire	C419	22-Nov-2019	F	Porcine endogenous retrovirus type C PCR	DNA	POSITIVE	25.85
3/30/22	Yorkshire	C429	25-Nov-2019	F	Porcine endogenous retrovirus type C PCR	Serum	Suspect	36.69
10/8/20	Landrace	C43	28-Nov-2019	M	Porcine endogenous retrovirus type C PCR	Buffy Coat	Neg	0
1/25/22	Yorkshire	C446	1-Dec-2019	M	Porcine endogenous retrovirus type C PCR	DNA	POSITIVE	29.6
2/1/22	Yorkshire	C448	7-Dec-2019	M	Porcine endogenous retrovirus type C PCR	Serum	POSITIVE	34.36
1/25/22	Yorkshire	C453	10-Dec-2019	F	Porcine endogenous retrovirus type C PCR	DNA	POSITIVE	30.1
3/30/22	Yorkshire	C454	13-Dec-2019	F	Porcine endogenous retrovirus type C PCR	Serum	Suspect	36.47
5/20/22	Yorkshire	C455	16-Dec-2019	F	Porcine endogenous retrovirus type C PCR	Serum	Neg	0
3/30/22	Yorkshire	C456	19-Dec-2019	F	Porcine endogenous retrovirus type C PCR	Serum	POSITIVE	31.94
1/25/22	Yorkshire	C457	22-Dec-2019	F	Porcine endogenous retrovirus type C PCR	DNA	POSITIVE	28.68
1/25/22	Yorkshire	C458	28-Dec-2019	F	Porcine endogenous retrovirus type C PCR	DNA	POSITIVE	29.53
2/1/22	Duroc	C497	3-Jan-2020	M	Porcine endogenous retrovirus type C PCR	Serum	Neg	0
2/1/22	Duroc	C505	6-Jan-2020	M	Porcine endogenous retrovirus type C PCR	Serum	Neg	0
1/25/22	Landrace	C51	9-Jan-2020	F	Porcine endogenous retrovirus type C PCR	DNA	Suspect	39.34
1/25/22	Crossbreed	C519	12-Jan-2020	F	Porcine endogenous retrovirus type C PCR	DNA	POSITIVE	28.29
6/17/22	Crossbreed	C521	15-Jan-2020	F	Porcine endogenous retrovirus type C PCR	Serum	Neg	0
6/17/22	Crossbreed	C543	18-Jan-2020	F	Porcine endogenous retrovirus type C PCR	Serum	Neg	0
6/17/22	Crossbreed	C544	21-Jan-2020	F	Porcine endogenous retrovirus type C PCR	Serum	Neg	0
3/30/22	Yorkshire	C558	24-Jan-2020	F	Porcine endogenous retrovirus type C PCR	Serum	Suspect	37.91
3/30/22	Yorkshire	C559	27-Jan-2020	F	Porcine endogenous retrovirus type C PCR	Serum	POSITIVE	35.56
3/30/22	Yorkshire	C561	30-Jan-2020	F	Porcine endogenous retrovirus type C PCR	Serum	Suspect	37.39
3/30/22	Yorkshire	C562	2-Feb-2020	F	Porcine endogenous retrovirus type C PCR	Serum	POSITIVE	34.24
3/30/22	Yorkshire	C564	5-Feb-2020	F	Porcine endogenous retrovirus type C PCR	Serum	POSITIVE	34.79
3/30/22	Landrace	C570	8-Feb-2020	F	Porcine endogenous retrovirus type C PCR	Serum	Suspect	36.93
3/30/22	Landrace	C571	11-Feb-2020	F	Porcine endogenous retrovirus type C PCR	Serum	Suspect	38.17
3/30/22	Landrace	C572	14-Feb-2020	F	Porcine endogenous retrovirus type C PCR	Serum	Suspect	36.32
3/30/22	Landrace	C573	17-Feb-2020	F	Porcine endogenous retrovirus type C PCR	Serum	POSITIVE	32.98
3/30/22	Landrace	C574	20-Feb-2020	F	Porcine endogenous retrovirus type C PCR	Serum	POSITIVE	33.34
3/30/22	Landrace	C575	23-Feb-2020	F	Porcine endogenous retrovirus type C PCR	Serum	Suspect	36.13
3/30/22	Landrace	C586	26-Feb-2020	F	Porcine endogenous retrovirus type C PCR	Serum	POSITIVE	32.32
3/30/22	Landrace	C587	29-Feb-2020	F	Porcine endogenous retrovirus type C PCR	Serum	POSITIVE	34.43
3/30/22	Landrace	C588	3-Mar-2020	F	Porcine endogenous retrovirus type C PCR	Serum	Suspect	38.35
3/30/22	Landrace	C589	6-Mar-2020	F	Porcine endogenous retrovirus type C PCR	Serum	Suspect	36.97
2/1/22	Landrace	C609	9-Mar-2020	M	Porcine endogenous retrovirus type C PCR	Serum	POSITIVE	33.03
2/1/22	Landrace	C610	12-Mar-2020	M	Porcine endogenous retrovirus type C PCR	Serum	Neg	0
2/1/22	Landrace	C611	15-Mar-2020	M	Porcine endogenous retrovirus type C PCR	Serum	Neg	0
2/1/22	Landrace	C612	18-Mar-2020	F	Porcine endogenous retrovirus type C PCR	Serum	POSITIVE	33.06
2/1/22	Landrace	C613	21-Mar-2020	F	Porcine endogenous retrovirus type C PCR	Serum	Neg	0
2/1/22	Landrace	C615	24-Mar-2020	F	Porcine endogenous retrovirus type C PCR	Serum	POSITIVE	28.94
2/1/22	Landrace	C616	27-Mar-2020	F	Porcine endogenous retrovirus type C PCR	Serum	POSITIVE	33.19
3/30/22	Landrace	C626	30-Mar-2020	F	Porcine endogenous retrovirus type C PCR	Serum	POSITIVE	34.14
3/30/22	Landrace	C627	2-Apr-2020	F	Porcine endogenous retrovirus type C PCR	Serum	POSITIVE	33.45
3/30/22	Landrace	C628	5-Apr-2020	F	Porcine endogenous retrovirus type C PCR	Serum	POSITIVE	34.08
3/30/22	Landrace	C629	8-Apr-2020	F	Porcine endogenous retrovirus type C PCR	Serum	POSITIVE	33.26
3/30/22	Landrace	C630	11-Apr-2020	F	Porcine endogenous retrovirus type C PCR	Serum	POSITIVE	35.45
3/30/22	Landrace	C631	14-Apr-2020	F	Porcine endogenous retrovirus type C PCR	Serum	POSITIVE	32.39
3/30/22	Yorkshire	C634	17-Apr-2020	M	Porcine endogenous retrovirus type C PCR	Serum	Neg	0
3/30/22	Yorkshire	C639	20-Apr-2020	F	Porcine endogenous retrovirus type C PCR	Serum	Neg	0
3/30/22	Yorkshire	C640	23-Apr-2020	F	Porcine endogenous retrovirus type C PCR	Serum	POSITIVE	35.83
3/30/22	Yorkshire	C641	26-Apr-2020	F	Porcine endogenous retrovirus type C PCR	Serum	POSITIVE	33.26
3/30/22	Yorkshire	C642	29-Apr-2020	F	Porcine endogenous retrovirus type C PCR	Serum	Neg	0
3/30/22	Yorkshire	C646	2-May-2020	F	Porcine endogenous retrovirus type C PCR	Serum	Neg	0
3/30/22	Yorkshire	C65	5-May-2020	F	Porcine endogenous retrovirus type C PCR	Serum	POSITIVE	30.56
4/12/22	Yorkshire	C650	8-May-2020	M	Porcine endogenous retrovirus type C PCR	Serum	POSITIVE	32.73
9/21/22	Crossbreed	C661	11-May-2020	F	Porcine endogenous retrovirus type C PCR	Serum	Suspect	38.06
9/30/22	Crossbreed	C672	17-May-2020	F	Porcine endogenous retrovirus type C PCR	Buffy Coat	POSITIVE	35.16
3/30/22	Yorkshire	C68	20-May-2020	F	Porcine endogenous retrovirus type C PCR	Serum	Suspect	38.01
5/20/22	Crossbreed	C685	23-May-2020	F	Porcine endogenous retrovirus type C PCR	Serum	POSITIVE	31.88
5/20/22	Crossbreed	C686	26-May-2020	F	Porcine endogenous retrovirus type C PCR	Serum	POSITIVE	32.53
5/20/22	Crossbreed	C687	29-May-2020	F	Porcine endogenous retrovirus type C PCR	Serum	POSITIVE	27.76
6/17/22	Landrace	C701	1-Jun-2020	M	Porcine endogenous retrovirus type C PCR	Serum	POSITIVE	33.14
6/17/22	Landrace	C702	4-Jun-2020	M	Porcine endogenous retrovirus type C PCR	Serum	POSITIVE	33.42
6/17/22	Landrace	C703	7-Jun-2020	M	Porcine endogenous retrovirus type C PCR	Serum	POSITIVE	34.07
6/17/22	Landrace	C704	10-Jun-2020	F	Porcine endogenous retrovirus type C PCR	Serum	Neg	0
6/17/22	Landrace	C705	13-Jun-2020	F	Porcine endogenous retrovirus type C PCR	Serum	Neg	0
6/17/22	Landrace	C706	16-Jun-2020	F	Porcine endogenous retrovirus type C PCR	Serum	POSITIVE	33.88
6/17/22	Landrace	C707	19-Jun-2020	F	Porcine endogenous retrovirus type C PCR	Serum	POSITIVE	32.01
6/17/22	Landrace	C708	22-Jun-2020	F	Porcine endogenous retrovirus type C PCR	Serum	POSITIVE	32.88
6/17/22	Landrace	C710	25-Jun-2020	M	Porcine endogenous retrovirus type C PCR	Serum	POSITIVE	33.84
6/17/22	Landrace	C711	28-Jun-2020	M	Porcine endogenous retrovirus type C PCR	Serum	POSITIVE	33.06
10/13/22	Yorkshire	C764	1-Jul-2020	F	Porcine endogenous retrovirus type C PCR	Serum	Suspect	38.4
10/13/22	Yorkshire	C765	4-Jul-2020	F	Porcine endogenous retrovirus type C PCR	Serum	Suspect	37.13
4/20/23	Yorkshire	C786	7-Jul-2020	F	Porcine endogenous retrovirus type C PCR	Blood	Neg	0
4/20/23	Yorkshire	C787	10-Jul-2020	F	Porcine endogenous retrovirus type C PCR	Blood	POSITIVE	33.93
3/15/23	Yorkshire	C834	13-Jul-2020	F	Porcine endogenous retrovirus type C PCR	Serum	Neg	0
3/15/23	Yorkshire	C835	16-Jul-2020	F	Porcine endogenous retrovirus type C PCR	Serum	POSITIVE	35.02
3/15/23	Yorkshire	C836	19-Jul-2020	F	Porcine endogenous retrovirus type C PCR	Serum	POSITIVE	33.93
3/15/23	Yorkshire	C837	22-Jul-2020	F	Porcine endogenous retrovirus type C PCR	Serum	Neg	0
3/15/23	Yorkshire	C838	25-Jul-2020	F	Porcine endogenous retrovirus type C PCR	Serum	POSITIVE	34.86
3/15/23	Yorkshire	C845	28-Jul-2020	F	Porcine endogenous retrovirus type C PCR	Serum	POSITIVE	35.05
3/15/23	Yorkshire	C847	31-Jul-2020	F	Porcine endogenous retrovirus type C PCR	Serum	POSITIVE	34.45
3/15/23	Yorkshire	C848	3-Aug-2020	F	Porcine endogenous retrovirus type C PCR	Serum	POSITIVE	33.72
3/15/23	Yorkshire	C849	6-Aug-2020	F	Porcine endogenous retrovirus type C PCR	Serum	POSITIVE	33.48
3/15/23	Yorkshire	C850	9-Aug-2020	F	Porcine endogenous retrovirus type C PCR	Serum	POSITIVE	34.16
3/15/23	Yorkshire	C855	12-Aug-2020	F	Porcine endogenous retrovirus type C PCR	Serum	Neg	0
3/15/23	Yorkshire	C856	15-Aug-2020	F	Porcine endogenous retrovirus type C PCR	Serum	Suspect	37.3
4/20/23	Yorkshire	C862	18-Aug-2020	F	Porcine endogenous retrovirus type C PCR	Blood	Suspect	36
4/20/23	Yorkshire	C863	21-Aug-2020	F	Porcine endogenous retrovirus type C PCR	Blood	Suspect	37.23
4/20/23	Yorkshire	C864	24-Aug-2020	F	Porcine endogenous retrovirus type C PCR	Blood	Suspect	37.05
4/20/23	Yorkshire	C868	27-Aug-2020	F	Porcine endogenous retrovirus type C PCR	Blood	Suspect	37.03
4/20/23	Yorkshire	C869	30-Aug-2020	F	Porcine endogenous retrovirus type C PCR	Blood	POSITIVE	34.72
4/20/23	Crossbreed	C891	2-Sep-2020	F	Porcine endogenous retrovirus type C PCR	Blood	Neg	0
4/20/23	Crossbreed	C892	5-Sep-2020	F	Porcine endogenous retrovirus type C PCR	Blood	Neg	0
4/20/23	Crossbreed	C893	8-Sep-2020	F	Porcine endogenous retrovirus type C PCR	Blood	Neg	0
4/20/23	Crossbreed	C894	11-Sep-2020	F	Porcine endogenous retrovirus type C PCR	Blood	Neg	0
4/12/22	Crossbreed	C949	14-Sep-2020	M	Porcine endogenous retrovirus type C PCR	Serum	POSITIVE	33.65
7/7/23	Yorkshire	C959	17-Sep-2020	F	Porcine endogenous retrovirus type C PCR	Serum	POSITIVE	31.63
7/7/23	Yorkshire	C960	20-Sep-2020	F	Porcine endogenous retrovirus type C PCR	Serum	POSITIVE	32.26
7/7/23	Yorkshire	C961	23-Sep-2020	F	Porcine endogenous retrovirus type C PCR	Serum	POSITIVE	30.75
7/7/23	Yorkshire	C962	26-Sep-2020	F	Porcine endogenous retrovirus type C PCR	Serum	POSITIVE	34

Supplementary Table 1: Screening of 121 pigs for PERV-C at the Unviersity of Minnesota Veterinary Diagnostic Lab (UMN VDL) using the primers developed by Martina et al. All results and cycle time values are summarized directly as reported by the UMN VDL. Results reported are positive are highlighted in red, suspect in yellow, and negative in green.
